# Development of a complex intervention for early integration of palliative home care into standard care for end-stage COPD patients: A Phase 0–I study

**DOI:** 10.1371/journal.pone.0203326

**Published:** 2018-09-19

**Authors:** Charlotte Scheerens, Kenneth Chambaere, Koen Pardon, Eric Derom, Simon Van Belle, Guy Joos, Peter Pype, Luc Deliens

**Affiliations:** 1 End-of-Life Care Research Group, Ghent University & Vrije Universiteit Brussel (VUB), Ghent, Belgium; 2 Department of Internal Medicine, Ghent University, Ghent, Belgium; 3 Department of Respiratory Medicine, Ghent University Hospital, Ghent, Belgium; 4 Department of Medical Oncology, Ghent University Hospital, Ghent, Belgium; 5 Department of Family Medicine and Primary Health Care, Ghent University, Ghent, Belgium; Nord University, NORWAY

## Abstract

**Background:**

Research suggests that palliative home care should be integrated early into standard care for end-stage COPD patients. Patients also express the wish to be cared for and to die at home. However, a practice model for early integration of palliative home care (PHC) into standard care for end-stage COPD has not been fully developed.

**Aim:**

To develop an intervention for early integration of PHC into standard care for end-stage COPD patients.

**Methods:**

We conducted a Phase 0–I study according to the Medical Research Council Framework for the development of complex interventions. Phase 0 aimed to identify the inclusion criteria and key components of the intervention by way of an explorative literature search of interventions, expert consultations, and seven focus groups with general practitioners and community nurses on perceived barriers to and facilitators of early integrated PHC for COPD. In Phase 1, the intervention, its inclusion criteria and its components were developed and further refined by an expert panel and two expert opinions.

**Results:**

Phase 0 resulted in identification of inclusion criteria and components from existing interventions, and barriers to and facilitators of early integration of PHC for end-stage COPD. Based on these findings, a nurse-led intervention was developed in Phase I consisting of training for PHC nurses in symptom recognition and physical therapy exercises for end-stage COPD, regular visits by PHC nurses at the patients’ homes, two information leaflets on self-management, a semi-structured protocol and follow-up plan to record the outcomes of the home visits, and integration of care by enabling collaboration and communication between home and hospital-based professional caregivers.

**Conclusion:**

This Phase 0-I trial succeeded in developing a complex intervention for early integration of PHC for end-stage COPD. The use of three methods in Phase 0 gave reliable data on which to base inclusion criteria and components of the intervention. The preliminary effectiveness, feasibility and acceptability of the intervention will be subsequently tested in a Phase II study.

## Background

Chronic Obstructive Pulmonary Disease (COPD) is one of the leading causes of death[1] and involves a progressive, inexorable functional decline and acute episodes of exacerbation[2]. End-stage COPD patients display symptoms including dyspnea, fatigue, anxiety and low mood, leading to a reduced quality of life[3]. Even with medical care, these symptoms impact heavily on daily activities, emotional and social functioning[3] while physical and psychosocial needs are inadequately addressed[4].

Integrating palliative care (PC) early into regular care could address these unmet needs and could have a positive impact on end-stage COPD patients[5,6]. Conversations about prognosis should be an integral part of care; strategies for professional caregivers to facilitate these discussions include being aware of the implications of the diagnosis, building a good relationship with the patient and starting the discussion of prognosis early in the disease course[7]. While patients with end-stage COPD often die in intensive care unit settings in hospital rather than at home[8], they actually prefer to be cared for at home[9] and to die at home[10]. If PC was provided at home it could improve quality of life[11,12], and increase the chances of dying at home[13].

Moreover, if palliative home care (PHC) was provided early enough it could avoid unnecessary hospital visits and admissions, overly aggressive care and excessive end-of-life-related medical costs[14]. Patients with end-stage COPD themselves express the need for integrated PHC as an addition to standard care[15]. However, implementing PHC early is not without its challenges because the unpredictability of the illness trajectory and survival time can complicate decisions about when to introduce it[5]. There are other challenges e.g. that some professional caregivers view PC for end-stage COPD as not valuable or believe that PC needs can be addressed by standard care alone[16].

Furthermore, research on implementing and testing early integrated PHC in clinical practice for end-stage COPD is fairly limited, with studies focusing either on PHC or on early PC, but not on both, except for one trial that tested acceptance of home support and integrated care among end-stage COPD patients[15]. Other studies and trials explored place of death and costs of medical care for COPD patients receiving PHC[13], early introduction of specialised PC for COPD[17,18], management of mainly one symptom i.e. breathlessness through PHC[19] or identification tools for end-stage COPD patients in need of proactive PC[20]. Interventions in cancer research have also tested some models of early and/or integrated PC/PHC demonstrating positive effects on quality of life and quality of care[21–25]. However, a practical model to implement early integration of PHC in standard home care for end-stage COPD patients is lacking; therefore, our study aim was to develop an evidence-based intervention supporting the early integration of PHC into standard care for end-stage COPD patients.

## Methods

### Study design

**Fig 1:**
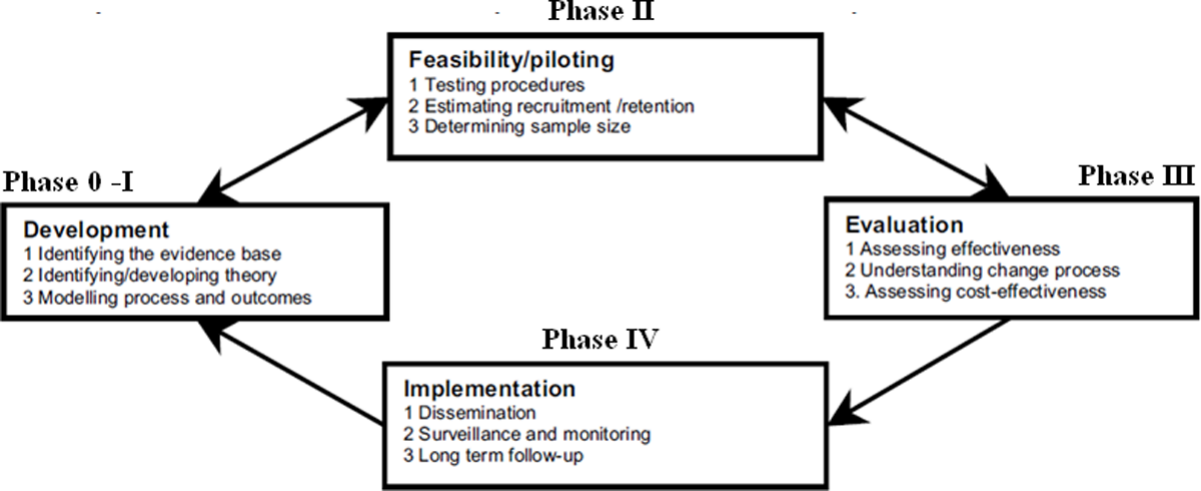
Medical Research Council framework for development and evaluation of complex interventions.

The intervention was developed using the Medical Research Council (MRC) framework for complex intervention design[26]. This framework provides multiples steps (from Phase 0 to Phase IV) for developing and evaluating complex interventions. The process may take different forms, with several Phases consisting of key functions and activities. The arrows indicate the main interactions between the Phases (Fig 1). Reporting is not shown as a separate activity, because it is regarded as an important element of each stage in the process[26]. This study consists of a Phase 0-I trial involving the identification and modelling of the inclusion criteria, core components of the intervention.

### Phase 0 –Identifying the evidence base and developing the theory

Three methods were used to obtain data for developing inclusion criteria and key components. Key components—the core of the intervention—are intended to be, and have been, positively associated with the outcomes that address identified needs[27,28].

The first method was an exploration of relevant literature, taking place between April 2015 and November 2016. This search identified published trial results and protocol papers of uncompleted trials on PC and/or symptom management for COPD. The used methodology was an explorative literature search. A search strategy was developed by CS and KC for PubMed. A combination of controlled vocabulary and free text words was used to search in titles and abstracts: COPD and intervention in combination with PC, early PC, general practitioners (GPs), and symptom management. The reference list of all identified studies was screened for additional relevant studies. A study was included if it reported: (1) Intervention results or an intervention protocol (the most recent results of one trial needed to be included), (2) Components related to PC or symptom management for end-stage COPD patients, and (3) Clear reference to inclusion criteria of participants. The eligibility of selected studies was independently assessed by CS and KC, and this selection was then revised by the research team. Appraisal was undertaken by CS and KC by critically reviewing all selected studies. No appraisal tool was used as the design was exploratory and not systematically.

The second method, undertaken between May and September 2016, was to consult experts for their views on possible inclusion criteria and components, based on their experiences and ideas about the future of PHC. 27 national and international experts from either clinical and/or research settings were selected for their research and/or clinical expertise in PC and/or COPD as a criterion. An extra criterion was stipulated for Belgian experts, namely their knowledge about the Belgian and Flemish healthcare context concerning care for COPD, as this was information we could not gather from the literature search and from international experts. By recruiting both experts in PC and/or in COPD we tried to obtain a sample of experts representing a wide range of experience related to the topic (maximum variation sampling). Other international and national experts in COPD and/or PC were recruited through professional contacts of experts we interviewed (snowball sampling). A topic guide in English and in Dutch can be seen in S1 Table.

Thirdly, we conducted focus groups between September 2015 and September 2016 to gain more insight into PHC practices, current standard care, barriers to be overcome and facilitators to promote early integration of PHC for end-stage COPD patients in Flanders, Belgium. This method complemented the previous methods with information about the specific Flemish context of the intervention. The methods of the focus groups are published elsewhere[29].

### Phase I: Modelling the intervention for clinical practice

In Phase I, the intervention was modelled[30]. Outcomes of the intervention were stipulated to be improvement in quality of life for end-stage COPD patients and in quality of care. The research team developed a first draft of a complex intervention, based on the results of Phase 0. The most common used and mentioned inclusion criteria and intervention components were selected, while also taking into account the research setting, the Flemish context, possibilities to replicate the intervention, feasibility and acceptability issues. Refinements to this draft were done by one expert panel and two individual expert opinions who further selected the best courses of action in order to enhance implementation. These Flemish experts identified possible implementation barriers which could occur and searched for solutions to overcome these barriers.

#### Flemish expert panel and expert opinions for implementation issues

The Flemish experts were selected for knowledge about COPD, either in clinical practice, in PHC or in social work. The expert panel and expert opinions were held in December 2016, of which the panel took one and a half hour, was audio taped and transcribed verbatim, while the expert opinions were via e-mail. The expert panel (n = 8) consisted of three GPs, a pulmonologist, a pulmonary nurse, a community nurse (CN) and a psychologist who works in a PHC team. Some of them had also been consulted for Phase 0 in the expert consultations or the focus groups. The two expert opinions were provided by a GP and a social worker. They all went through the draft of the intervention for improvements and adaptations. The inclusion criteria were also revised on implementation issues by a well-experienced pulmonologist. The obtained data were categorised for each inclusion criterion and component and analysed within the research team to finalise the intervention. A flow diagram of the methods used from Phase 0 to I is given in Table 1.

**Table 1:** Use of the theory and modelling Phase from the Medical Research Council framework.

Definition	Steps undertaken
(1) Phase 0 –theory	
(1.1) Identifying the evidence base by carrying out an explorative literature search	(i) Reviewed existing interventions on palliative care and symptom management for end-stage COPD patients on their design, components, inclusion criteria, and results on outcomes. (ii) Reviewed existing intervention protocols on palliative care and symptom management for end-stage COPD patients on their design, components, inclusion criteria, and chosen outcomes.
(1.2) Identifying international and national insights on possible inclusion criteria and core components by carrying out expert consultations	(i) Interviewed 21 experts on their view about successful interventions for early integration of palliative home care for end-stage COPD, based on the inclusion criteria and components we identified in the explorative literature search, and on other components the experts identified during the consultations.
(1.3) Identifying insights on the Flemish context of early integrated palliative home care for end-stage COPD, which could not be derived from 1.1 and 1.2	(i) Conducted three focus group conversations with general practitioners and four with community nurses on barriers and facilitators for early integration of palliative home care for end-stage COPD patients in Flanders.
(2) Phase I–modelling the intervention	
(2.1) Selecting inclusion criteria by using a pragmatic approach based on the critical consideration of the research team, using the results from Phase 0 and taking into account the Flemish clinical practice context, the research setting, feasibility and acceptability issues	(i) Linked all results on inclusion criteria from the different methods in Phase 0 and analysedreisbu them. (ii) Selected the most common used or positively mentioned inclusion criteria from both literature and expert consultations. (iii) Sorted the inclusion criteria on their relevance for the intervention, taking into account the Flemish clinical practice and palliative home care context by also consulting the obtained results from the focus groups on the facilitator: trigger moments.
(2.2) Selecting intervention components by using a pragmatic approach based on the critical consideration of the research team, using the results from Phase 0 and taking into account the Flemish clinical practice context, the research setting, feasibility and acceptability issues, and possibilities for replicating it. Outcomes of the intervention should be improvement of quality of life for end-stage COPD patients and quality of care.	(i) Linked all results on key components from all methods used in Phase 0 and compared the results. (ii) Identified the most common used or positively mentioned components from both literature and expert consultations. (iii) Compared the most common used or positively mentioned components from the literature search and the expert consultations and sorted them on their relevance for the intervention. This was done by taking into account their applicability in the Flemish context as we reviewed the identified barriers and facilitators from the focus group study in relation to these components. (iiii) Selected and designed five key components in a first draft of the intervention, using combinations of components from previous interventions, material from previous projects for COPD and new developed components by the research team members themselves.
(2.3) Identifying implementation issues concerning the chosen inclusion criteria and components specific for the Flemish context	(i) Reviewed the most common inclusion and exclusion criteria identified in Phase 0 by a pulmonologist with long experience in clinical practice for end-stage COPD on implementation issues and feasibility and adapted several criteria for better implementation chances. (ii) Consulted the involved palliative home care team on feasibility and acceptability issues of the selected components. (iii) Consulted an expert panel and two expert opinions on their views, comments and suggestions of the first draft of the intervention, focusing on implementation, feasibility and acceptability issues.
(2.4) Finalising the intervention model	The research team analysed the obtained remarks from the pulmonologist, involved palliative home care team, expert panel and expert opinions and adjusted the intervention’s inclusion criteria and components in a final draft.

### Ethical aspects

The research protocol and topic guides for the focus groups were approved by the Ethics Committee of Ghent University Hospital (Reference: 2016/0171).A signed informed consent was obtained from each participant in the focus groups and the expert consultations, panel and opinions. Anonymity was assured by preventing the participants being identified from the transcripts.

## Results

### Phase 0 –Identifying the evidence base and developing theory

#### Exploration of relevant literature

**Fig 2:**
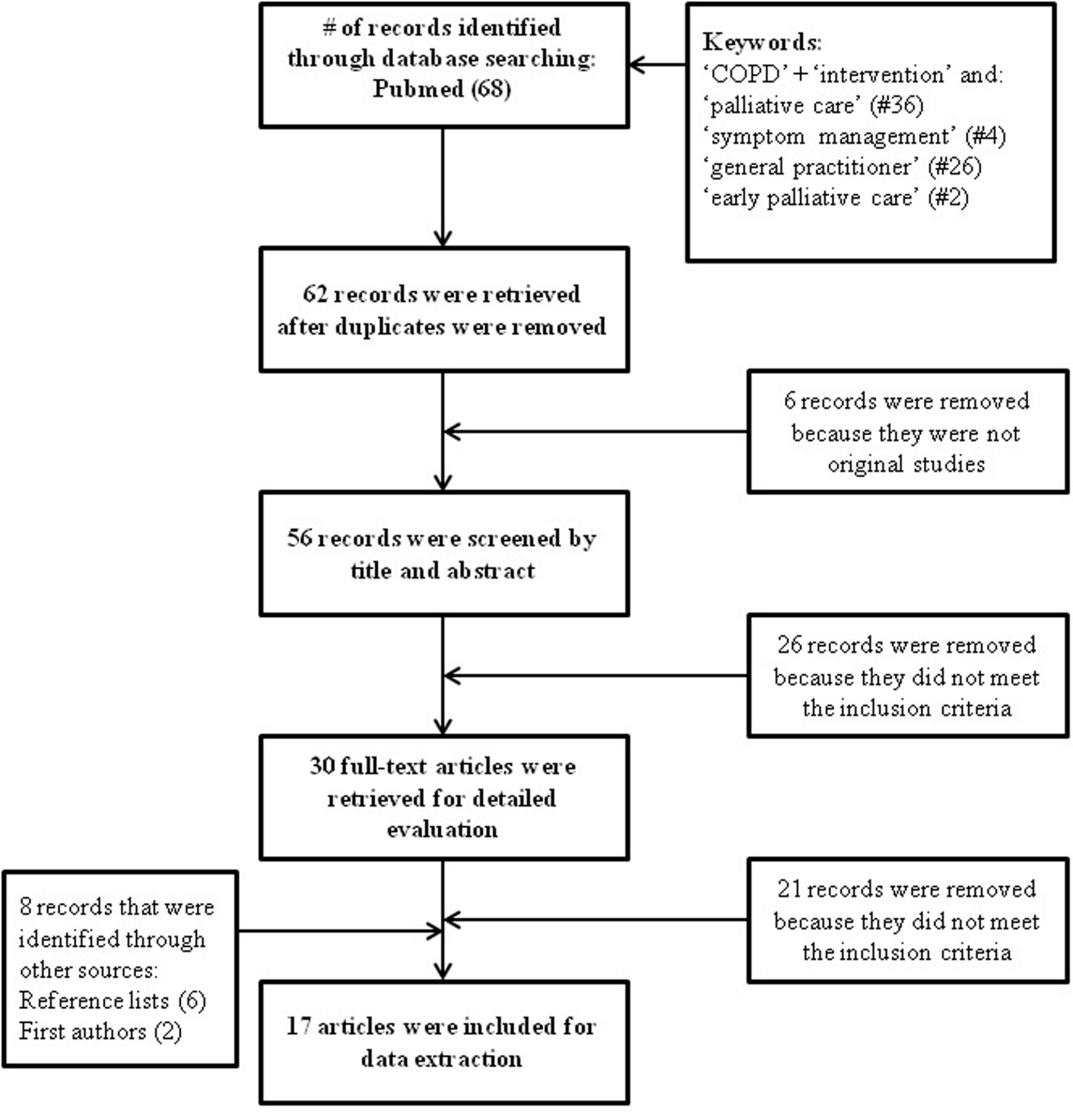
Full electronic search strategy.

From the explorative electronic database search 68 records were identified. After removal of duplicates and irrelevant reports, the title and abstract of 58 records was screened and 30 full-text articles were retrieved for detailed evaluation. Contact with the first authors and a search in reference lists of included articles yielded eight records. Seventeen articles met all inclusion criteria and were included for data-extraction and quality assessment (Fig 2). Eleven were intervention studies and six were intervention protocols.

The most commonly used inclusion criteria in the studies were hospitalisation for an exacerbation (recently, one or more times in the last year), end-stage dyspnea (according to the Medical Research Council Dyspnea score) and lung function scores (mostly GOLD III/IV) (Table 2).

**Table 2:** Inclusion criteria of interventions and intervention protocols on palliative care and symptom management for end-stage COPD patients based on explorative literature search.

Inclusion criteria	Used x times in explored studies
Hospitalisation for an exacerbation (recently OR 1–2 times in last year(s))	10
End-stage dyspnea (Medial Research Council dyspnea Scale score 5)	5
End-stage COPD (mostly GOLD III/IV)	5
FEV1 (airflow limited)	3
Age	3
Smoking habits (ex-smoker or intending to quit/current or former smoker)	3
Hypercapnia/hypoxemia	2
BMI (<21)	2
Free of exacerbation last month	2
Housebound	1
Inhalation therapy	1
Domiciliary oxygen/ home ventilation	1
Surprise question (will die within one year or readmission within 8 weeks)	1
Comorbidity	1
Hospital Anxiety and Depression Scale score > = 8	1
Visit for pulmonary follow-up	1

The most used key components in the analysed studies were *(1) advance care planning*: training GPs[31] and respiratory nurses[32], or testing whether advance care planning affected the end-stage COPD patient’s decision-making about future preferences[33]; *(2) respiratory rehabilitation*: simple home and intensive hospital-based pulmonary rehabilitation programmes[34], the involvement of respiratory health workers[35] or the integration of respiratory services with standard care therapy[6]; *(3) specialised PC*: specialised PC added to standard care[18,20]; *(4) training professional caregivers*: training GPs in early identification of end-stage COPD patients in need of PC, training on communication about preferences and written advance directives[31] or training on inhalation techniques[36]; *(5) educating end-stage COPD patients*: educating end-stage COPD patients about identification and treatment of exacerbations[37], decision-making in end-of-life care[38] or cognitive restructuring where patients learn to interpret physical and psychological symptoms about anxiety and dyspnea[39], and *(6) improving self-management of end-stage COPD patients*: using telemonitoring for symptom reporting[40], developing a breathlessness service for managing breathlessness[19,41] or using an action plan with a holistic assessment of physical, psychological, social and spiritual/existential needs[42]. A broader overview of the key components derived from the explorative literature search can be seen in S2 Table, and in-detail examination of it in S3 Table.

#### Expert consultations

Of the 27 experts invited for consultation, 21 accepted the invitation. Nine were from Belgium, of whom six had expertise in PC and three in COPD. Twelve were from other countries, of whom seven were PC experts, two were experts in COPD, and three were experts in both PC and COPD. Experts were pulmonary specialists, a physiotherapist, experienced researchers, policymakers, people working in a PHC unit and GPs. (Table 3).

**Table 3:** Characteristics of consulted experts.

Expert number	Profession	Country	Expertise
1	Palliative home care head nurse Coordinator palliative home care team	Belgium	Palliative care
2	Palliative home care nurse	Belgium	Palliative care
3	General practitioner Palliative home care physician Scientific researcher	Belgium	Palliative care
4	General practitioner Scientific researcher	Belgium	Palliative care
5	General practitioner Scientific researcher	Belgium	COPD
6	General practitioner Scientific researcher	Belgium	Palliative care
7	Pulmonary physician Scientific researcher	Belgium	COPD
8	Pulmonary physiotherapist	Belgium	COPD
9	Respiratory physician Scientific researcher	Switzerland	Palliative care COPD
10	General practitioner Palliative home care physician	Belgium	Palliative care
11	Respiratory physician Scientific researcher	Australia	COPD
12	Respiratory physician	Australia	Palliative care COPD
13	Respiratory physician Scientific researcher	Spain	Palliative care
14	Palliative care physician Scientific researcher	Spain	Palliative care
15	Researcher social sciences	Germany	Palliative care
16	Palliative care physician Researcher medical science	Canada	Palliative care
17	Member of a Scientific Institute Professor in medicine	United Kingdom	Palliative care
18	Member of an expertise centre in Palliative Care Researcher in pain and palliative medicine	Netherlands	Palliative care
19	Scientific researcher	Netherlands	Palliative care
20	Scientific researcher	United Kingdom	Palliative care COPD
21	Respiratory and sleep physician	Australia	COPD

The experts considered inclusion and exclusion criteria by suggesting which criterion indicated PHC needs, and which not. They mentioned positive (facilitators) and negative (barriers) comments for each criterion. The most common mentioned inclusion criteria were: after admission in hospital for exacerbation (eight experts in favor), depending on lung function test (eight in favor, but also six not in favor), depending of functioning of the patient (four in favor) and being housebound (three in favor) (Table 4).

Possible components were discussed: advance care planning, psychosocial support and symptom control were recommended respectively by fourteen, eleven and eleven experts, although advance care planning was also seen as less suitable by nine and the benefits of psychosocial support were questioned by four. Increasing the patient’s knowledge and disease insight and providing pulmonary rehabilitation were each recommended by nine experts but barriers were also mentioned by respectively two and four. Eight experts stressed the importance of incorporating self-management into the intervention, while two reported barriers to that. Lastly, six experts saw possibilities for the involvement of informal caregivers in a PHC intervention, and three questioned it (Table 4). S4 Table provides an in-detail description of the mentioned barriers and facilitators for inclusion criteria and components.

**Table 4:** Summary of possible inclusion criteria and components for early integration of palliative home care according to expert consultations.

**Inclusion criteria**	Facilitators mentioned by experts*						Barriers mentioned by experts*					
	A	B	C	D	E	**Total**	A	B	C	D	E	**Total**
After admission in hospital for exacerbation			12, 22	14, 16, 17, 19, 20	21	**8**		4	22			**2**
Functioning of the patient			22	14, 16	10	**4**						**0**
Depending on lung function test		4	12, 22	14, 15, 16, 18, 19		**8**	9	4		15, 16, 19, 20		**6**
Depending of social context						**0**				16		**1**
Opinion of caregiver	7					**1**						**0**
Being housebound			12	14, 16		**3**			22			**1**
Oxygen dependency			12, 22			**2**						**0**
**Component**	Facilitators mentioned by experts*						Barriers mentioned by experts*					
	A	B	C	D	E	**Total**	A	B	C	D	E	**Total**
Advance Care Planning	5	1, 2, 3, 4, 11	12, 22	15, 17, 18, 19, 20	13	**14**	5	3, 11	12, 22	15, 20	13, 21	**9**
Involvement of informal caregivers	9		12, 22	15, 16	21	**6**	9		22	16		**3**
Knowledge and disease-insight	9	1, 2, 11	22	15, 17, 18	10	**9**				15	10	**2**
Pulmonary rehabilitation	9	1, 2, 3, 4	22	17, 18	21	**9**	9			17, 18	21	**4**
Psychosocial support	5	1, 2, 6		14, 15, 16, 17, 18	10, 21	**11**		3		14, 15, 16		**4**
Self-management		1, 2	22	15, 17, 18	10, 21	**8**				15, 16		**2**
Symptom control		1, 2, 3, 4, 11	12	15, 17, 18	10, 21	**11**						**0**

*The results are presented in following order: **A**: Belgian experts in COPD; **B**: Belgian experts in palliative care; **C**: Foreign experts in COPD; **D**: Foreign experts in palliative care;

**E**: Foreign experts in COPD and palliative care. The numbers in these boxes (with exception of the total numbers) represent the experts’ numbers from Table 3.

#### Focus groups

Three focus groups with GPs (n = 8, n = 8, n = 10) and four with CNs (n = 4, n = 7, n = 5, n = 12) were held. The categories of barriers related to early integration of PHC were (1) Unpredictable exacerbations and death in COPD and invisible deterioration of functioning; (2) Perceived patient attitudes such as a lack of disease insight and resistance towards care; (3) Professional caregiver practices with a lack of a coherent and proactive plan, insufficient experience and a negative view of PHC for end-stage COPD; (4) Not enough focus on knowledge and advantages of PHC and PC for end-stage COPD in professional caregivers’ basic and continuing education; (5) Healthcare and PHC system characteristics: too short consultations, insufficient coordination between hospital and home care, and a reimbursement system for PHC that is based on life expectancy; and (6) Communication: a lack of and unclear communication between professional caregivers about further care possibilities for end-stage COPD patients, and a lack of clear information about PHC between professional caregivers and their patients.

The categories of facilitators were (1) Trigger moments to start talking about early integration of PHC such as after hospitalisation, after a couple of exacerbations, when an end-stage COPD patient becomes oxygen-dependent or becomes housebound; (2) Involvement of informal caregivers in early integrated PHC for COPD; (3) Information about the advantages of early integrated PHC for end-stage COPD in professional caregivers’ education; (4) Including advance care planning as a part of healthcare and PHC systems and (5) Communication: enhancing communication between professional caregivers by installing a care coordinator, and between professional caregivers and end-stage COPD patients by explaining better and in a practical way early integrated PHC. The elaborate results are published elsewhere[29].

### Phase I: Modelling process and outcomes

#### Inclusion criteria

The most common used or mentioned inclusion criteria from literature and expert consultations were cross referenced to Flemish and clinical implementation issues. Lung function tests (GOLD III and GOLD IV[43]) were selected as a basic inclusion criterion to identify the end-stage of COPD. Next, as recommendations from GOLD[43] and the results in Phase 0 also stipulated that lowering of functioning and frequent exacerbations are evenly important predictors of end-stage COPD, we decided to combine lung function tests with two (in case of GOLD III) or one (in case of GOLD IV) criteria/criterion representing frequent and severe exacerbations (three or more hospitalisations for COPD in the past three years) or lowering of functioning (oxygen-dependent, COPD Assessment Test-scale ≥25, Medical Research Council dyspnea-scale of 4, intubation or non-invasive ventilation in the past year, low Body Mass Index or heart failure New York Heart Association of 3). By designing the criteria in this way, we tried to incorporate the diverse ways end-stage COPD could occur. The selected exclusion criteria reflect a patient able to participate in the study such as region of residence, Dutch speaking and no or low cognitive impairment. We also excluded active cancers as patients would otherwise have a higher chance of referral to PHC because of their cancer diagnosis (Table 5).

**Table 5:** Final selection of inclusion and exclusion criteria.

Inclusion criteria	Exclusion criteria
GOLD III (cf. GOLD 2017[43]) and ≥ 2 of following criteria OR GOLD IV (cf. GOLD 2017[43]) and ≥1 of following criteria: ➢ Oxygen-dependent[44] ➢ Three or more hospitalisations for COPD in the past three years ➢ COPD Assessment Test -scale ≥25[43] ➢ Medical Research Council Scale Dyspnea 4 ➢ Intubation in the past year ➢ Non-invasive ventilation in the past year ➢ Body Mass Index ≤18 ➢ New York Hart Association- scale of 3	Patients living outside the region Ghent-Eeklo, Flanders, Belgium (where the intervention will take place) Patients in the last days of life (bedbound and/or semi-comatose and/or take only fluid and/or no longer able to take oral drugs[18]) Patients with cognitive impairment: Mini Mental status Examination ≤23 at the day of inclusion of patients[45] Lung cancer diagnosis Patients with active cancer Patients who are not living at home any more Patients with no knowledge of Dutch Patients with a general practitioner already involved in this study for an intervention/control group patient

#### Components

The five final components were developed by combining all results from Phase 0. Predefined outcomes for the intervention were improving quality of life for end-stage COPD patients and quality of care. Therefore, we prioritised the most common used or mentioned components from the literature and expert consultations: advance care planning, pulmonary rehabilitation, patients’ knowledge and disease-insight and symptom control. The results also suggested to include the involvement of informal caregivers, psychosocial support and self-management as a component. Furthermore, the results from the focus group study revealed that advance care planning, training professional caregivers, increasing knowledge about COPD and PHC for patients and involving informal caregivers could enhance early integrated PHC. Almost all these components were included in the first draft of the design, representing a holistic PHC approach[42].

However, the final selection of components took into account the Flemish context of PHC, feasibility issues for the involved PHC team and for the research setting, replication possibilities for other studies and implementation issues in clinical practice. Pulmonary rehabilitation was therefore excluded as a component on its own, as this was not feasible for the involved PHC team of the intervention. Also, advance care planning and involving informal caregivers were combined in one component. As PHC teams in Flanders already offer advance care planning and support for informal caregivers as standard PHC, we chose to focus the component more on a systematic reporting tool about these subjects, as this still lacking in their current care management.

After the content of the intervention was decided, we modelled and further operationalised it into five concrete components using elements from previous interventions and projects as well new ideas from the research team. Further adjustments were made by the expert panel and expert opinions. The components are: (1) Training of the PHC team in care for end-stage COPD patients, (2) Monthly home visits by a PHC nurse, (3) Information leaflets, (4) A semi-structured protocol for home visits, and (5) Integration of care between involved professional caregivers.

*Component 1—Training of the PHC team in care for end-stage COPD patients*: Training in knowledge and care for patients with end-stage COPD will be provided to the nurses of the PHC team, who expressed the need for this. The PHC team will also learn to work with the semi-structured protocol and the information leaflets. The expert panel confirmed the need for training the team and agreed on the topics (see infra) included in the training. One expert advised the inclusion of information on the value of physical activity and dietary advice as many end-stage COPD patients suffer from nutritional deficiencies and low muscle mass. The research team decided to include this recommendation. The topics of training will be:

- Providing information on the disease trajectory of COPD and the symptom burden for end-stage COPD patients
- Recognising and managing an exacerbation
- Providing information about the role of exercise, breathing exercises, coping with the disease and self-management skills
- Learning to work with the semi-structured protocol that will be used during home visits
- Learning to work with the information leaflets ‘Breathing and saving energy’ and ‘Preventing and coping with complaints’

*Component 2—Home visits by a PHC nurse*: Home visits by a PHC nurse are already part of PHC as usual to support the patient and their informal caregivers. However, end-stage COPD patients are not receiving PHC visits systematically in Flanders and if so, it is mainly in the terminal phase[46]. In Phase 0, it became clear from the literature[47] that end-stage COPD patients need more systematic support for their symptoms and concerns, and not only in the terminal phase. Therefore, systematic home visits by a PHC nurse are incorporated as a component with a minimum frequency of one per month over a period of six months in total, which can be increased if more follow-up would be needed. This was seen as appropriate by the expert panel.

*Component 3—Information leaflets*: In current PHC practice in Flanders, tools for patients’ self-management, knowledge and disease insight are insufficiently used, although this could vary among PHC teams. In contrast, both the literature[19,41] and our expert consultations provided evidence for the benefits of appending self-management tools in PHC for end-stage COPD patients. Therefore, information leaflets on self-management of COPD symptoms were selected as an component. The leaflets will be offered to the patient during PHC visits. The expert panel recommended that Flemish leaflets should be used to improve comprehensibility for Flemish people, but as there were none in existence at the time, Dutch-translated leaflets from the ‘Living well with COPD’ Canadian project, originally developed in English, were chosen[48]. This project is a Canadian learning method for COPD patients and their families. It focuses on raising awareness and knowledge about COPD and on teaching patients possible coping mechanisms, breathing exercises, knowledge and use of medication. The Dutch-translated leaflets were developed for patients living in the Netherlands and contain therefore information e.g. brand names of medication only available in the Netherlands. Following the expert panel’s advise, we replaced this with a list of medication available in Belgium. Also, some words used in the Dutch-translated leaflets are not used in Flemish, but as we considered the general content very clear for Flemish speaking patients, this was left unchanged. However, we will analyse this possible bias in the feasibility and acceptability study from the Phase II intervention.

*Component 4 –Semi-structured protocol during home visits*: Currently, most PHC teams in Flanders do not use a standardised registration document listing and reporting all possible topics of care and support covered during home visits. PHC nurses also lack knowledge about the specific symptom burden in end-stage COPD. We therefore developed a semi-structured protocol for reporting about a pre-defined list of COPD-related care and support management topics during home visits. The structure of the protocol is inspired by the interventions of Weber et al[17], Buckingham et al[42] and Vanbutsele et al[23] and contains nine focus areas, representing a holistic PHC approach: disease-insight and coping, symptom management (flagged by completing The Dutch version of the Edmonton Symptom Assessment Scale (ESAS)), care planning, support for informal caregivers, psychosocial support, spiritual support, other non-predefined support e.g. financial, practical and administrative, coordination of care and an action plan (Table 6). The expert panel recommended changing some words from the Dutch ESAS into Flemish in order to improve interpretation. They also added ‘tightness in the chest’ next to ‘breathlessness’ as the former referred to another symptom than the latter.

**Table 6:** Focus areas in the semi-structured protocol for the palliative home care nurse.

Focus area	Explanation
Disease insight and coping	Anamnesis of the disease and the patient Listening to the patient’s experience of his/her dyspnea Information about dyspnea and COPD (through conversation and info-leaflets) and medication (if needed)
Symptom management	Standard care package of the palliative home care team Assessment of symptoms (ESAS)
Care planning	Values and wishes of the patient for the future Preferences for end-of-life care (for example about hospitalisations) Living will/advance directive
Support for those close to the person who is dying (if needed)	Identification of those closest to the person Assessment of their needs Providing available resources if needed
Psychosocial support	Assessment of needs (psychological, social, financial, administrative, activities of daily living) Providing available resources if needed If needed, referral to other professional caregivers or social workers if patients agrees
Spiritual/existential support	Assessment of needs (how he/she see the future, who he/she get existential/spiritual support from, if there are particular worries) Providing available support
Other support	Assessment of other concerns or needs (for example: practical needs related to housing) Follow-up of problems if help can be given
Coordination of care	Listing all involved professional caregivers, their function, treatment goals and interventions Involving professional caregivers in the follow-up of the patient and their vision on further care Communication about coordination of care with the involved general practitioner
Action plan	Listing agreed actions the patient can undertake to tackle certain problems, identified in one or more of the other focus areas Patient, general practitioner, community nurse, physiotherapist and pulmonologist receive an overview of these actions after each visit

*Component 5 –Integration of care between involved professional caregivers*: Current PHC in Flanders does not use standard reporting or communication procedures between PHC nurses and other professional caregivers, except for regular contact with the GP, who instigates PHC. As seen in Phase 0, the focus groups stressed the need for a care coordinator to install a proactive care plan as conflicting treatment between professional caregivers often occurs. Consequently, the component ‘integration of care’ will systematise report mechanisms and communication between the PHC nurses and involved professional caregivers. With the agreement of the patient, the PHC nurse will send a report of the semi-structured protocol with the action plan and the overview of the coordination after each home visit to the GP, CN, physiotherapist and pulmonologist. The GPs will be particularly involved as the PHC nurse will always contact them if further action or care is needed and if important changes in the health status of the patient would occur. An overview of how the final components were derived from the Phase 0 results can be seen in Table 7.

**Table 7:** Describing components of an intervention to integrate palliative home care early in standard care for end-stage COPD patients.

**Component 1: Training of the palliative home care team in care for end-stage COPD patients**	
**Explanation of the component**	**Barriers and facilitators addressed by the component**
**Five nurses of the palliative home care team who will execute the intervention will follow a half-day training course on care for COPD patients. This is to prepare them for the intervention with COPD patients as the majority of those they currently support are cancer patients. A pulmonologist and a pulmonary physiotherapist/smoking cessation consultant from Ghent University hospital will give this part of the training. Another part will consist of learning to use the intervention tools (information leaflets and semi-structured protocol: see infra under component 3 and component 4) as the palliative home care nurses are not experienced in using them. This will be given by the executive researcher of the study, a sociologist from Ghent University.**	➢ No experience in clinical practice with palliative care for COPD (focus group—barrier) ➢ Not enough focus on knowledge and advantages of palliative care for COPD in basic and continuing education (focus group- barrier) ➢ More focus on early integration of PHC for COPD and concrete implementation in clinical practice in education for professional caregivers (focus group facilitator) ➢ Training professional caregivers on early identification of patients in need of palliative care and structuring advance care planning (literature)
**Component 2: monthly home visits by a palliative home care nurse**	
**Explanation of the component**	**Barriers and facilitators addressed by the component**
**After the general practitioner has been brought into contact with the palliative home care nurse and the patient has been discharged at least two weeks from hospital (if the inclusion happened during hospitalisation), the patient will meet the palliative home care nurse during a first home visit where the palliative home care nurse will introduce himself/herself and explain what the palliative home care team can do for the patient during the intervention. Following this introductory visit, the palliative home care nurse will plan home visits at least once a month for a period of six months in total, the length of the study. If more adequate follow-up is needed, a higher frequency of visits will be given.**	➢ Specialised palliative care consultations integrated with standard care (literature) ➢ Palliative status for palliative home care is based on predictability of death (focus group-barrier) ➢ Palliative reimbursement of palliative home care is restricted to 3 months (focus group- barrier) ➢ Not enough time during consultations to start talking about palliative care and further care (focus group–barrier) ➢ Not discussing palliative care (needs) in detail during consultations with the end-stage COPD patient (focus group-barrier) ➢ Professional caregivers fear talking about palliative home care because of the patient’s reaction (focus group-barrier) ➢ Difficulties for professional caregivers to talk about palliative care needs with their end-stage COPD patients (focus group-barrier)
**Component 3: Information leaflets**	
**Explanation of the component**	**Barriers and facilitators addressed by the component**
**A palliative home care nurse will give, during one or more of the home visits, two information leaflets to the patient. The information leaflets have the goal to raise awareness and knowledge about COPD and to inform the patient on possible coping mechanisms. These information leaflets are Dutch-translated from the originally English leaflets and are part of the Canadian ‘Living well with COPD’ project, which is a learning method for patients and their families. Titles of the information leaflets are ‘Prevent and coping with complaints’ and ‘Breathing and saving energy’. The palliative home care nurse will explain the information leaflets during visit 1 and visit 2 (and in case of lack of time during visit 3) as good as possible to the patient and teach the patient how to use it. It is recommended to start with ‘Prevent and coping with complaints’ and then ‘breathing and saving energy’.**	➢ Educating patients with COPD (literature) ➢ Self-management of patients with COPD (literature) ➢ Not understanding the severity of the disease or realizing the possibility of death (focus group- barrier) ➢ Denial of the severity of the disease (focus group- barrier) ➢ Inform patients clearly and firmly about their disease and the future (focus group–facilitator) ➢ Knowledge and disease insight (expert consultations)
**Component 4: Semi-structured protocol during home visits**	
**Explanation of the component**	**Barriers and facilitators addressed by the component**
**During each visit, the palliative home care nurse will use a semi-structured protocol to structure the visit and report about it (see Table 6). This semi-structured protocol will tackle the following areas: disease-insight and coping, symptom management, care planning, support for informal caregivers, psychosocial support (psychological, financial and administrative), spiritual support, other non-predefined support, an action plan and coordination of care. During every visit, the palliative home care nurse will fill in the semi-structured protocol, which will contain fill-in boxes for the nine focus areas, a box to note down actions and how many minutes of their visit were spent on each area. An action plan and a care plan will be included as well and will be updated during each visit.**	➢ Communication between caregiver and patient (focus group–barrier) ◦ Not discussing palliative care (needs) in detail during consultations with the patient ◦ Difficulties for professional caregivers to talk about end-of-life preferences and palliative care needs with patients ◦ Patient-relative relationship can prevent communication about palliative care ◦ Professional caregivers fear talking about palliative care because of the patient’s reaction ➢ Increase knowledge about advantages of palliative home care for informal caregivers of patients with COPD (focus group–facilitator) ➢ Start advance care planning as a standard procedure for all severe COPD patients living at home (FG—facilitator) ➢ Communication between caregiver and the patient (focus group–facilitator) ◦ Talking about practical matters can help the doctor start talking about palliative care ◦ Better explanation of the term palliative home care can help acceptance by patient ➢ Advance care planning as a part of palliative care (literature) ➢ Self-management of patients with COPD (literature) ➢ Advance care planning as a component of palliative care trials (expert consultations) ➢ Psychosocial support (expert consultations) ➢ Symptom control (expert consultations) ➢ Involvement of those close to the patient (expert consultations)
**Component 5: Integration of care between involved professional caregivers**	
**Explanation of the component**	**Barriers and facilitators addressed by the component**
**The palliative home care team, responsible general practitioner, community nurse, physiotherapist and pulmonologist systematically receive, if the patient agrees, a report of every visit with a summary of the assessments and agreed actions. The palliative home care nurse sends this report directly to them through e-mail or post.** **The general practitioner(and if needed other relevant professional caregivers such as the community nurses) will be contacted by the palliative home care nurse to develop the care plan for the patient. If needed, any medical interventions by other professional caregivers can be coordinated together with the responsible general practitioner.**	➢ Lack of a coherent and proactive care plan (focus group barrier) ◦ No cooperation between professional caregivers involved at home ◦ Conflicting therapy and treatment between professional caregivers ➢ Interprofessional communication (focus group barrier) ◦ Not knowing each other well enough for proper communication ◦ Unclear who takes initiative to introduce PHC to the patient ◦ Not understanding each other’s messages ➢ Communication between professional caregivers: appoint a care coordinator who facilitates the care transition to palliative home care (focus group–facilitator)

## Discussion

### Summary of the results

In this article we described the development and modelling of an intervention of early integrated palliative home care (PHC) for end-stage Chronic Obstructive Pulmonary Disease (COPD) patients. Predefined outcomes were improvement in quality of life for end-stage COPD patients and quality of care. Phase 0 resulted in the identification of possible inclusion criteria, components, barriers to and facilitators for early integration of PHC for end-stage COPD. Based on these findings, in Phase I, a nurse-led intervention was developed with inclusion criteria representing PHC needs and a decline in functioning. Five components were modelled: (1) Training on symptom recognition and physical therapy exercises for the involved PHC team; (2) Regular home visits by PHC nurses; (3) Two information leaflets on self-management of COPD; (4) A semi-structured protocol to record the outcomes of the PHC visits and (5) Integration of care by encouraging collaboration and communication between involved professional caregivers in primary and secondary care.

### Strengths and weaknesses

A key strength was conducting a Phase 0–I study according to the Medical Research Council (MRC)-framework, as this provided a high-quality structured and phased process towards the development of a complex intervention[28]. This is confirmed by previous interventions on end-of-life care and advance care planning[6,27]. To our knowledge this is also the first nurse-led intervention exploring early integration of PHC for end-stage COPD, by combining several components with a holistic PHC approach.

One limitation is that we did not directly consult the perspectives of end-stage COPD patients and their informal caregivers in Phase 0 whereas the primary focus was in fact to gain insight into care possibilities for early integration of PHC. The views of end-stage COPD patients on PHC have been captured in previous research[15], although without taking into account the specific Flemish context. However, interviews with half of the patients and informal caregivers from the intervention group will be conducted in the Phase II evaluation study, where their perspectives on the intervention components and on possible improvements for a Phase III intervention trial will be explored.

Another limitation is that we did not included pulmonary rehabilitation as a separate component despite recommendations from expert consultations and literature, in the form of physical therapy sessions[34] or for breathlessness relief[6]. This was decided because pulmonary rehabilitation is formally not included in Flemish PHC services and the involved PHC nurses who will execute the intervention are not experienced with it. However, we included rehabilitation techniques and breathing exercises in the component ‘training for the PHC team’ and the PHC team will refer the patient to a pulmonary physiotherapist if needed. Furthermore, in the Phase II evaluation study, the necessity of incorporating it as a component in future interventions will be evaluated.

### Comparison with existing literature

The component ‘semi-structured protocol’ was developed using combined elements from three previous interventions. The components of Weber et al[18] were used for shaping the content of the focus areas: symptom control, spiritual/existential, psychosocial and support for informal caregivers, knowledge about the disease and coordination of care. The components of Buckingham’s intervention[42] added a concrete self-management tool in the form of an action plan. From Vanbutsele’s et al [23] intervention we took the structure of the semi-structured protocol document, with indications of how long (in minutes) a PHC nurse will discuss one topic. This led to a semi-structured protocol which encompasses a complete care support programme.

When comparing the intervention with study results in a recent review[5] about PC and symptom management for COPD, similarities and differences occurred. The review summarised current evidence on how symptoms and concerns in COPD could be addressed using PC interventions, and also deduced potential models for integrative working e.g. symptom-triggered services, short-term integrated PC, advanced COPD clinics and integrated respiratory care services (pulmonary rehabilitation, early supported discharge, hospital at home etc.). The findings indicated that these models could be triggered by indications for PC such as complex troublesome symptoms, a hospitalisation, change in place of residence, acute inpatient care for respiratory failure and the commencement of oxygen therapy. In the meantime, respiratory medicine and primary care could be offered simultaneously to the patient[5]. These suggested models focused mainly on managing specific symptoms such as breathlessness; which differed from the holistic focus of our intervention. A similarity is that the review supported early integration of PC with standard respiratory services. In our intervention pulmonologists will also receive monthly reports from the PHC nurse and will be able to co-decide, with the PHC nurse, about certain actions for the patient.

### Implications for research, education and practice

Future research could focus on evaluating results of PHC interventions for end-stage COPD, and on analysing procedures and difficulties in enrolling and developing these interventions. Phase II interventions should also report more feasibility and acceptability results of participants, and could actively involve the latter in the development and evaluation phases. Currently, there is a lack of knowledge about all these aspects. Not only is the number of interventions on PHC for end-stage COPD low, published interventions also often fail to report study procedures, which impedes replication of interventions in other contexts. More reviews like Maddocks et al[5], that proved its usefulness for in-sight information about trials and studies about PC for end-stage COPD, could provide this lacking information.

Furthermore, our results imply that more knowledge about care and support for end-stage COPD is needed for PHC nurses, but also for general practitioners (GPs) and community nurses. Emphasis on this topic should be provided in basic education with the possibility of learning skills in clinical practice. Further education on PHC should also include more disease specific support, for example learning breathing exercises for end-stage COPD, and should thus not be limited to care and support for cancer patients. In clinical practice for end-stage COPD, the roles of involved professional caregivers could be re-evaluated, by training PHC nurses better in providing pulmonary rehabilitation or teaching physiotherapists better in care for end-stage COPD. Early PHC for end-stage COPD, opposed to PHC given in the final stages of life or PHC for cancer, might also benefit from the involvement of pulmonary physiotherapists and psychologists besides PHC nurses.

### Conclusion

This Phase 0-I process succeeded in developing a complex intervention for early integration of PHC into standard care for end-stage COPD patients. Three methods in Phase 0 gave reliable data with inclusion criteria and components for an intervention focused on holistic PHC. The feasibility, acceptability and preliminary effectiveness of the intervention will be subsequently tested in a Phase II study.

## Supporting information

S1 Table Topic guide expert consultations in English and in Dutch. (DOCX)

S2 Table Key components of existing interventions and intervention protocols on palliative care and symptom management for end-stage COPD patients based on explorative literature search. (DOCX)

S3 Table Explorative literature search on interventions and intervention protocols. (DOCX)

S4 Table Overview of barriers and facilitators on inclusion criteria and components from the expert consultations. (DOCX)

## References

[1] Mannino DM. COPD: epidemiology, prevalence, morbidity and mortality, and disease heterogeneity. Chest [Internet]. 2002 5 [cited 2016 Nov 29];121(5 Suppl):121S–126S. Available from: http://www.ncbi.nlm.nih.gov/pubmed/12010839 12010839 10.1378/chest.121.5_suppl.121s

[2] Clinical indications for noninvasive positive pressure ventilation in chronic respiratory failure due to restrictive lung disease, COPD, and nocturnal hypoventilation—a consensus conference report. Chest [Internet]. 1999 8 [cited 2016 Nov 29];116(2):521–34. Available from: http://www.ncbi.nlm.nih.gov/pubmed/10453883 10453883 10.1378/chest.116.2.521

[3] McSweeny AJ, Grant I, Heaton RK, Adams KM, Timms RM. Life quality of patients with chronic obstructive pulmonary disease. Arch Intern Med [Internet]. 1982 3 [cited 2016 Nov 29];142(3):473–8. Available from: http://www.ncbi.nlm.nih.gov/pubmed/7065785 7065785

[4] Gore JM, Brophy CJ, Greenstone MA. How well do we care for patients with end stage chronic obstructive pulmonary disease (COPD)? A comparison of palliative care and quality of life in COPD and lung cancer. Thorax [Internet]. 2000 12 [cited 2017 Feb 6];55(12):1000–6. Available from: http://www.ncbi.nlm.nih.gov/pubmed/11083884 10.1136/thorax.55.12.1000 11083884 PMC1745647

[5] Maddocks M, Lovell N, Booth S, Man WD-C, Higginson IJ. Palliative care and management of troublesome symptoms for people with chronic obstructive pulmonary disease. Lancet (London, England) [Internet]. 2017 9 2 [cited 2017 Oct 10];390(10098):988–1002. Available from: http://www.ncbi.nlm.nih.gov/pubmed/28872031 10.1016/S0140-6736(17)32127-X 28872031

[6] Higginson IJ, Bausewein C, Reilly CC, Gao W, Gysels M, Dzingina M, et al An integrated palliative and respiratory care service for patients with advanced disease and refractory breathlessness: A randomised controlled trial. Lancet Respir Med [Internet]. 2014;2(12):979–87. Available from: 10.1016/S2213-2600(14)70226-7 25465642

[7] Halliwell J, Mulcahy P, Buetow S, Bray Y, Coster G, Osman LM. GP discussion of prognosis with patients with severe chronic obstructive pulmonary disease: a qualitative study. Br J Gen Pract [Internet]. 2004 12 [cited 2017 Nov 23];54(509):904–8. Available from: http://www.ncbi.nlm.nih.gov/pubmed/15588534 15588534 PMC1326107

[8] Cohen J, Beernaert K, Van den Block L, Morin L, Hunt K, Miccinesi G, et al Differences in place of death between lung cancer and COPD patients: a 14-country study using death certificate data. npj Prim Care Respir Med [Internet]. 2017 12 3 [cited 2017 Mar 17];27(1):14 Available from: http://www.ncbi.nlm.nih.gov/pubmed/28258277 10.1038/s41533-017-0017-y 28258277 PMC5434782

[9] Bereza BG, Troelsgaard Nielsen A, Valgardsson S, Hemels ME, Einarson TR. Patient preferences in severe COPD and asthma: a comprehensive literature review. Int J Chron Obstruct Pulmon Dis [Internet]. 2015;10:739–44. Available from: http://www.pubmedcentral.nih.gov/articlerender.fcgi?artid=4399696&tool=pmcentrez&rendertype=abstract 10.2147/COPD.S82179 25914530 PMC4399696

[10] Gomes B, Calanzani N, Gysels M, Hall S, Higginson IJ. Heterogeneity and changes in preferences for dying at home: a systematic review. BMC Palliat Care [Internet]. 2013 2 15 [cited 2017 Feb 14];12(1):7 Available from: http://bmcpalliatcare.biomedcentral.com/articles/10.1186/1472-684X-12-7 23414145 10.1186/1472-684X-12-7 PMC3623898

[11] Gomes B, Calanzani N, Higginson IJ. Benefits and Costs of Home Palliative Care Compared With Usual Care for Patients With Advanced Illness and Their Family Caregivers. JAMA [Internet]. 2014 3 12 [cited 2017 May 29];311(10):1060 Available from: http://www.ncbi.nlm.nih.gov/pubmed/24618968 10.1001/jama.2014.553 24618968

[12] Singer AE, Goebel JR, Kim YS, Dy SM, Ahluwalia SC, Clifford M, et al Populations and Interventions for Palliative and End-of-Life Care: A Systematic Review. J Palliat Med [Internet]. 2016 9 [cited 2017 Sep 29];19(9):995–1008. Available from: http://www.ncbi.nlm.nih.gov/pubmed/27533892 10.1089/jpm.2015.0367 27533892 PMC5011630

[13] Enguidanos SM, Cherin D, Brumley R. Home-Based Palliative Care Study. J Soc Work End Life Palliat Care [Internet]. 2005 10 24 [cited 2017 Oct 23];1(3):37–56. Available from: http://www.ncbi.nlm.nih.gov/pubmed/17387068 10.1300/J457v01n03_04 17387068

[14] Brian Cassel J, Kerr KM, McClish DK, Skoro N, Johnson S, Wanke C, et al Effect of a Home-Based Palliative Care Program on Healthcare Use and Costs. J Am Geriatr Soc [Internet]. 2016 11 [cited 2017 Jun 23];64(11):2288–95. Available from: http://www.ncbi.nlm.nih.gov/pubmed/27590922 10.1111/jgs.14354 27590922 PMC5118096

[15] Damps-Konsta?ska I, Werachowska L, Krakowiak P, Kaczmarek M, Cynowska B, G?recka D, et al Acceptance of home support and integrated care among advanced COPD patients who live outside large medical centers. Appl Nurs Res [Internet]. 2016 8 [cited 2017 Jun 23];31:60–4. Available from: http://www.ncbi.nlm.nih.gov/pubmed/27397820 10.1016/j.apnr.2015.12.003 27397820

[16] Scheerens C, Beernaert K, Pype P, Cohen J, Deliens L, Chambaere K. Comparing the use and timing of palliative care services in COPD and lung cancer: a population-based survey. Eur Respir J [Internet]. 2018 5 24 [cited 2018 May 30];51(5):1702405 Available from: http://www.ncbi.nlm.nih.gov/pubmed/29794123 10.1183/13993003.02405-2017 29794123

[17] Weber C, Stirnemann J, Herrmann FR, Pautex S, Janssens J-P, Horton R, et al Implementing a palliative care trial in advanced COPD: a feasibility assessment (the COPD IMPACT study). BMC Palliat Care [Internet]. 2013;13(47):67–73. Available from: http://www.pubmedcentral.nih.gov/articlerender.fcgi?artid=3546432&tool=pmcentrez&rendertype=abstract 10.1089/jpm.2012.0285 PMC3546432 23317322

[18] Weber C, Stirnemann J, Herrmann FR, Pautex S, Janssens J-P. Can early introduction of specialized palliative care limit intensive care, emergency and hospital admissions in patients with severe and very severe COPD? a randomized study. BMC Palliat Care. 2014;13(47):1–7. 25927907 10.1186/1472-684X-13-47 PMC4448287

[19] Farquhar MC, Prevost AT, McCrone P, Brafman-Price B, Bentley A, Higginson IJ, et al The clinical and cost effectiveness of a Breathlessness Intervention Service for patients with advanced non-malignant disease and their informal carers: mixed findings of a mixed method randomised controlled trial. Trials [Internet]. 2016;17(1):185 Available from: http://www.ncbi.nlm.nih.gov/pubmed/27044249%5Cn http://www.pubmedcentral.nih.gov/articlerender.fcgi?artid=PMC4820876 27044249 10.1186/s13063-016-1304-6 PMC4820876

[20] Duenk RG, Heijdra Y, Verhagen SC, Dekhuijzen RPNR, Vissers KCP, Engels Y. PROLONG: a cluster controlled trial to examine identification of patients with COPD with poor prognosis and implementation of proactive palliative care. BMC Pulm Med [Internet]. 2014;14(1):54 Available from: http://www.pubmedcentral.nih.gov/articlerender.fcgi?artid=3995742&tool=pmcentrez&rendertype=abstract 24690101 10.1186/1471-2466-14-54 PMC3995742

[21] Temel JS, Greer JA, El-Jawahri A, Pirl WF, Park ER, Jackson VA, et al Effects of Early Integrated Palliative Care in Patients With Lung and GI Cancer: A Randomized Clinical Trial. J Clin Oncol [Internet]. 2017 3 10 [cited 2017 Mar 15];35(8):834–41. Available from: http://www.ncbi.nlm.nih.gov/pubmed/28029308 10.1200/JCO.2016.70.5046 28029308 PMC5455686

[22] Zimmermann C, Swami N, Krzyzanowska M, Hannon B, Leighl N, Oza A, et al Early palliative care for patients with advanced cancer: a cluster-randomised controlled trial. Lancet [Internet]. 2014 5 17 [cited 2018 Jan 25];383(9930):1721–30. Available from: http://www.ncbi.nlm.nih.gov/pubmed/24559581 10.1016/S0140-6736(13)62416-2 24559581

[23] Vanbutsele G, Pardon K, Van Belle S, Surmont V, De Laat M, Colman R, et al Effect of early and systematic integration of palliative care in patients with advanced cancer: a randomised controlled trial. Lancet Oncol [Internet]. 2018 2 [cited 2018 Feb 5];0(0). Available from: http://linkinghub.elsevier.com/retrieve/pii/S1470204518300603 10.1016/S1470-2045(18)30060-3 29402701

[24] Bakitas MA, Tosteson TD, Li Z, Lyons KD, Hull JG, Li Z, et al Early Versus Delayed Initiation of Concurrent Palliative Oncology Care: Patient Outcomes in the ENABLE III Randomized Controlled Trial. J Clin Oncol [Internet]. 2015 5 1 [cited 2018 Jun 1];33(13):1438–45. Available from: http://www.ncbi.nlm.nih.gov/pubmed/25800768 10.1200/JCO.2014.58.6362 25800768 PMC4404422

[25] Temel JS, Greer JA, Muzikansky A, Gallagher ER, Admane S, Jackson VA, et al Early Palliative Care for Patients with Metastatic Non–Small-Cell Lung Cancer. N Engl J Med [Internet]. 2010 8 19 [cited 2016 Nov 29];363(8):733–42. Available from: http://www.nejm.org/doi/abs/10.1056/NEJMoa1000678 20818875 10.1056/NEJMoa1000678

[26] Medical Research Council. Medical Research Council: MRC framework for development and evaluation of RCTs for complex interventions to improve health. London; 2000.

[27] De Vleminck A, Houttekier D, Deliens L, Vander Stichele R, Pardon K. Development of a complex intervention to support the initiation of advance care planning by general practitioners in patients at risk of deteriorating or dying: a phase 0–1 study. BMC Palliat Care [Internet]. 2016 2 11 [cited 2017 Nov 10];15:17 Available from: http://www.ncbi.nlm.nih.gov/pubmed/26868650 10.1186/s12904-016-0091-x 26868650 PMC4750213

[28] Campbell M, Fitzpatrick R, Haines A, Kinmonth AL, Sandercock P, Spiegelhalter D, et al Framework for design and evaluation of complex interventions to improve health. BMJ [Internet]. 2000 9 16 [cited 2016 Nov 29];321(7262):694–6. Available from: http://www.ncbi.nlm.nih.gov/pubmed/10987780 10987780 10.1136/bmj.321.7262.694 PMC1118564

[29] Scheerens C, Deliens L, Van Belle S, Joos G, Pype P, Chambaere K. “A palliative end-stage COPD patient does not exist”: a qualitative study of barriers to and facilitators for early integration of palliative home care for end-stage COPD. npj Prim Care Respir Med [Internet]. 2018 12 20 [cited 2018 Jun 21];28(1):23 Available from: http://www.nature.com/articles/s41533-018-0091-9 10.1038/s41533-018-0091-9 29925846 PMC6010468

[30] Craig P, Dieppe P, Macintyre S, Michie S, Nazareth I, Petticrew M, et al Developing and evaluating complex interventions: the new Medical Research Council guidance. BMJ [Internet]. 2008 9 29 [cited 2018 Jun 13];337:a1655 Available from: http://www.ncbi.nlm.nih.gov/pubmed/18824488 10.1136/bmj.a1655 18824488 PMC2769032

[31] Thoonsen B, Vissers K, Verhagen S, Prins J, Bor H, van Weel C, et al Training general practitioners in early identification and anticipatory palliative care planning: a randomized controlled trial. BMC Fam Pract [Internet]. 2015;16(1):126 Available from: http://www.pubmedcentral.nih.gov/articlerender.fcgi?artid=4578268&tool=pmcentrez&rendertype=abstract 26395257 10.1186/s12875-015-0342-6 PMC4578268

[32] Houben CHM, Spruit M a, Wouters EFM, Janssen DJ a. A randomised controlled trial on the efficacy of advance care planning on the quality of end-of-life care and communication in patients with COPD: the research protocol. BMJ Open [Internet]. 2014;4(1):e004465 Available from: http://www.pubmedcentral.nih.gov/articlerender.fcgi?artid=3902375&tool=pmcentrez&rendertype=abstract 10.1136/bmjopen-2013-004465 24384905 PMC3902375

[33] Teno J, Lynn J, Wenger N, Phillips RS, Murphy DP, Connors AF Jr., et al Advance directives for seriously ill hospitalized patients: Effectiveness with the patient self-determination act and the SUPPORT intervention. J Am Geriatr Soc [Internet]. 1997;45(4):500–7. Available from: http://www.embase.com/search/results?subaction=viewrecord&from=export&id=L27169228%5Cn http://limo.libis.be/resolver?&sid=EMBASE&issn=00028614&id=doi:&atitle=Advance+directives+for+seriously+ill+hospitalized+patients%3A+Effectiveness+with+the+patient+self- 9100721 10.1111/j.1532-5415.1997.tb05178.x

[34] Guell MR, de Lucas P, Galdiz JB, Montemayor T, Rodriguez Gonzalez-Moro JM, Gorostiza A, et al [Home vs hospital-based pulmonary rehabilitation for patients with chronic obstructive pulmonary disease: a Spanish multicenter trial]. Arch Bronconeumol. 2008;44(10):512–8. 19006630

[35] Cockcroft A, Bagnall P, Heslop A, Andersson N, Heaton R, Batstone J, et al Controlled trial of respiratory health worker visiting patients with chronic respiratory disability. Br Med J [Internet]. 1987;294(6566):225–8. Available from: http://www.scopus.com/inward/record.url?eid=2-s2.0-0023066589&partnerID=40&md5=3e93d1fa907c923524dc10325a85b276 3101821 10.1136/bmj.294.6566.225 PMC1245234

[36] Leiva-Fernández J, Vázquez-Alarcón RL, Aguiar-Leiva V, Lobnig-Becerra M, Leiva-Fernández F, Barnestein-Fonseca P. Efficacy of an educational intervention in primary health care in inhalation techniques: study protocol for a pragmatic cluster randomised controlled trial. Trials [Internet]. 2016;17(1):144 Available from: http://www.trialsjournal.com/content/17/1/144 10.1186/s13063-016-1269-5 26988095 PMC4794820

[37] Randomized a, Trial C, Fan VS, Gaziano JM, Lew R, Bourbeau J, et al Original Research A Comprehensive Care Management Program to Prevent Chronic Obstructive Pulmonary Disease Hospitalizations. 2012;156(10). 10.7326/0003-4819-156-10-201205150-00003 22586006

[38] Horton R, Rocker G, Dale A, Young J, Hernandez P, Sinuff T. Implementing a palliative care trial in advanced COPD: a feasibility assessment (the COPD IMPACT study). J Palliat Med [Internet]. 2013 1 [cited 2017 Jan 30];16(1):67–73. Available from: http://online.liebertpub.com/doi/abs/10.1089/jpm.2012.0285 23317322 10.1089/jpm.2012.0285 PMC3546432

[39] Bove DG, Overgaard D, Lomborg K, Lindhardt BØ, Midtgaard J. Efficacy of a minimal home-based psychoeducative intervention versus usual care for managing anxiety and dyspnoea in patients with severe chronic obstructive pulmonary disease: a randomised controlled trial protocol. BMJ Open [Internet]. 2015;5(7):e008031 Available from: http://www.pubmedcentral.nih.gov/articlerender.fcgi?artid=4499678&tool=pmcentrez&rendertype=abstract 10.1136/bmjopen-2015-008031 26152326 PMC4499678

[40] Ho T-W, Huang C-T, Chiu H-C, Ruan S-Y, Tsai Y-J, Yu C-J, et al Effectiveness of Telemonitoring in Patients with Chronic Obstructive Pulmonary Disease in Taiwan-A Randomized Controlled Trial. Sci Rep [Internet]. 2016;6(March):23797 Available from: http://www.nature.com/articles/srep23797 27029815 10.1038/srep23797 PMC4814821

[41] Booth S, Moffat C, Farquhar M, Higginson IJ, Burkin J. Developing a breathlessness intervention service for patients with palliative and supportive care needs, irrespective of diagnosis. J Palliat Care [Internet]. 2011 [cited 2017 Dec 21];27(1):28–36. Available from: http://www.ncbi.nlm.nih.gov/pubmed/21510129 21510129

[42] Buckingham S, Kendall M, Ferguson S, MacNee W, Sheikh A, White P, et al HELPing older people with very severe chronic obstructive pulmonary disease (HELP-COPD): mixed-method feasibility pilot randomised controlled trial of a novel intervention. NPJ Prim care Respir Med. 2015;25(November 2014):15020 10.1038/npjpcrm.2015.20 26028347 PMC4532154

[43] Global Initiative for Chronic Obstructive Lung Disease (GOLD). From the Global Strategy for the Diagnosis, Management and Prevention of COPD [Internet]. 2017. Available from: http://goldcopd.org

[44] Gex G, Janssens J-P. [Long-term oxygen therapy]. Rev Med Suisse [Internet]. 2007 11 21 [cited 2016 Nov 29];3(134):2646, 2648–50, 2652–4. Available from: http://www.ncbi.nlm.nih.gov/pubmed/18159698 18159698

[45] Folstein MF, Folstein SE, McHugh PR. &quot;Mini-mental state&quot;. A practical method for grading the cognitive state of patients for the clinician. J Psychiatr Res [Internet]. 1975 11 [cited 2016 Nov 29];12(3):189–98. Available from: http://www.ncbi.nlm.nih.gov/pubmed/1202204 1202204 10.1016/0022-3956(75)90026-6

[46] Scheerens, C, Beernaert, K, Pype, P, Cohen, J, Deliens, L, Chambaere K (forthcoming). Comparing the use and timing of palliative care services in chronic obstructive pulmonary disease (COPD) and lung cancer: a population-based survey. 10.1183/13993003.02405-2017 29794123

[47] Duenk RG, Heijdra Y, Verhagen SC, Dekhuijzen RP, Vissers KC, Engels Y. PROLONG: a cluster controlled trial to examine identification of patients with COPD with poor prognosis and implementation of proactive palliative care. BMC Pulm Med [Internet]. 2014 12 2 [cited 2017 Oct 17];14(1):54 Available from: http://bmcpulmmed.biomedcentral.com/articles/10.1186/1471-2466-14-54 24690101 10.1186/1471-2466-14-54 PMC3995742

[48] the Montreal Chest Institute of the McGill University Health Centre. Living well with COPD [Internet]. [cited 2016 Feb 2]. Available from: http://www.livingwellwithcopd.com/en/about.html

