# Supplementary material for: Development of a complex intervention for early integration of palliative home care into standard care for end-stage COPD patients: A Phase 0–I study
Source: PLoS One. 2018 Sep 19;13(9):e0203326. doi: 10.1371/journal.pone.0203326 (PMC6145576; doi:10.1371/journal.pone.0203326)
Supplement: S1 Table — (DOCX) [file pone.0203326.s001.docx]

**S1 Table: Topic guide expert consultations in English and in Dutch**

| **Topic guide in English** | **Topic guide in Dutch** |
| --- | --- |
| - Introduction and explanation of the goal of the intervention: improving quality of life for people with end-stage COPD by early integration palliative home care - What is your affinity with the study topic and how would you address this in clinical practice? - What is the added value of early integrated palliative care for end-stage COPD? - How can professional caregivers decide when early integrated palliative care should be given for end-stage COPD? For example using trigger moments and inclusion criteria from literature? - How could the current problems with palliative home care for people with end-stage COPD be better addressed (through involvement of palliative home care teams)? - How could some solutions help improve quality of life for people with end-stage COPD? - Which professional caregiver could play a role in the improvement? - Asking their opinion about components used in literature: psychosocial support, symptom management, - How could early integration of palliative home care be implemented in standard care for people with end-stage COPD using which components in interventions? | - Introductie: Uitleg over mijn onderzoek en waarop we onze interventie zullen toespitsen: nl. het verbeteren van de levenskwaliteit van de patiënt met vergevorderde COPD en het zorgkader voor COPD. Dit door het integreren van vroegtijdige palliatieve zorg in de reguliere thuiszorg - Vragen naar hun affiniteit met het onderwerp en hoe zij dat zouden zien in de praktijk - Vragen over de zin van (vroegtijdige) palliatieve zorg bij COPD patiënten - Vragen naar welke triggers/inclusiecriteria vanuit de literatuur er bestaan voor de zorgverlener om te bepalen wanneer (vroegtijdige) palliatieve zorg kan ingeschakeld worden bij COPD - Hoe kunnen de huidige problemen voor COPD patiënten beter geadresseerd worden (door betrekken van palliatieve thuiszorg)? - Welke oplossingen kunnen levenskwaliteit verbeteren? - Welke rol zouden betrokken zorgverleners kunnen spelen in deze verbetering? - Welke interventiecomponenten kunnen gebruikt worden om tijdige integratie van palliatieve thuiszorg te introduceren in standaardzorg voor COPD? - Welke inclusiecriteria kunnen gebruikt worden om tijdige integratie van palliatieve thuiszorg te introduceren |
