# Supplementary material for: Development of a complex intervention for early integration of palliative home care into standard care for end-stage COPD patients: A Phase 0–I study
Source: PLoS One. 2018 Sep 19;13(9):e0203326. doi: 10.1371/journal.pone.0203326 (PMC6145576; doi:10.1371/journal.pone.0203326)
Supplement: S2 Table — (DOCX) [file pone.0203326.s002.docx]

**S2 Table: Key components of existing interventions and intervention protocols on palliative care and symptom management for end-stage COPD patients based on explorative literature search**

| **Key component** | **Explanation** |
| --- | --- |
| Advance care planning | Incorporating advance care planning was done by training general practitioners in proactive care planning^1^ or by testing the effectiveness of written advance directives on decision-making about future preferences, where health caregivers received information about each patient’s and surrogate’s preferences in the medical record^2^. A study trained respiratory nurses in structured advance care during 2-day sessions, focusing on reflection of patient’s goals, values and beliefs, understanding current and future medical situations, possible treatments, outcomes, and communication skills^3^. |
| Pulmonary rehabilitation and respiratory services | Guëll^4^ compared home-based and hospital based pulmonary rehabilitation where the patients attended two informative sessions about the disease and four physical therapy sessions. The home-based patients then performed at home low intensity exercises without supervision, while the hospital-based patients carried out a structured exercise programme. Cockcroft^5^ evaluated monthly visits to COPD patients by a respiratory nurse, who gave education and support focused on health according to individual needs. They followed a model identifying problems in activities of daily living and setting goals to increase independence in these activities. Higginson tested a breathlessness support service, being a short-term, single point of access service integrating PC, respiratory medicine, physiotherapy, and occupational therapy^6^. |
| Specialised palliative care | The study of Weber^7^ integrated early palliative care consultations (one per month for one year by a palliative care professional) into standard care, focusing on symptom management, understanding of illness and coping with the disease, anticipation, relatives support, social support, spiritual support, coordination of the health professionals. The intervention of Duenk^8^ let patients meet with a specialised palliative care team (who received special training for palliative care in COPD) within one week after enrolment and at least monthly thereafter in the outpatients setting for at least one year or until death, in the presence of the main informal caregiver. |
| Trained professional caregivers | Some interventions involved training general practitioners, such as a five hour course on early identification of patients in need of PC and on structuring anticipatory PC planning, an individual coaching session by phone with a physician specialised in PC, and peer group sessions on patient-general practitioner communication regarding the initiation of PC^1^. Another protocol provided training for general practitioners on educating patients to use the right inhalation techniques with their inhalers^9^. |
| Educating COPD patients | An intervention by Fan et al^10^The patient was given education on COPD over four individual and one group sessions, an action plan for identification and treatment of exacerbations, and scheduled proactive telephone calls for case management^11^. Another trial incorporated the Living Well with COPD programme and an educational module on end-of-life care and decision making in an intervention which was delivered in the patient’s home over four to eight weeks^12^. Lastly, a study is currently testing the impact of a psycho-educative session (focused on cognitive restructuring) in the patient’s home in combination with a telephone booster session where patients learned to interpret and react to physical and psychological symptoms that are related to dyspnea and associated anxiety^13^. |
| Self-management of patients with COPD | One trial used telemonitoring as a way for patients to report their symptoms daily using an electronic diary^14^. Another focused on managing breathlessness by developing a breathlessness intervention service which included among other things a relaxation CD, a hand-held fan and a wellbeing journal for increasing personal wellbeing ^15,16^, while Buckingham developed a respiratory nurse-led intervention with an action plan on proactive holistic assessment of physical, psychological, social and spiritual needs^17^. |

**Reference list**

1. Thoonsen B, Vissers K, Verhagen S, et al. Training general practitioners in early identification and anticipatory palliative care planning: a randomized controlled trial. *BMC Fam Pract*. 2015;16(1):126. doi:10.1186/s12875-015-0342-6.

2. Teno J, Lynn J, Wenger N, et al. Advance directives for seriously ill hospitalized patients: Effectiveness with the patient self-determination act and the SUPPORT intervention. *J Am Geriatr Soc*. 1997;45(4):500-507. doi:10.1111/j.1532-5415.1997.tb05178.x.

3. Houben CHM, Spruit M a, Wouters EFM, Janssen DJ a. A randomised controlled trial on the efficacy of advance care planning on the quality of end-of-life care and communication in patients with COPD: the research protocol. *BMJ Open*. 2014;4(1):e004465. doi:10.1136/bmjopen-2013-004465.

4. Guell MR, de Lucas P, Galdiz JB, et al. [Home vs hospital-based pulmonary rehabilitation for patients with chronic obstructive pulmonary disease: a Spanish multicenter trial]. *Arch Bronconeumol*. 2008;44(10):512-518.

5. Cockcroft A, Bagnall P, Heslop A, et al. Controlled trial of respiratory health worker visiting patients with chronic respiratory disability. *Br Med J*. 1987;294(6566):225-228. http://www.scopus.com/inward/record.url?eid=2-s2.0-0023066589&partnerID=40&md5=3e93d1fa907c923524dc10325a85b276.

6. Higginson IJ, Bausewein C, Reilly CC, et al. An integrated palliative and respiratory care service for patients with advanced disease and refractory breathlessness: A randomised controlled trial. *Lancet Respir Med*. 2014;2(12):979-987. doi:10.1016/S2213-2600(14)70226-7.

7. Weber C, Stirnemann J, Herrmann FR, Pautex S, Janssens J-P. Can early introduction of specialized palliative care limit intensive care, emergency and hospital admissions in patients with severe and very severe COPD? a randomized study. *BMC Palliat Care*. 2014;13(47):1-7. doi:10.1186/1472-684X-13-47.

8. Duenk RG, Heijdra Y, Verhagen SC, Dekhuijzen RP, Vissers KC, Engels Y. PROLONG: a cluster controlled trial to examine identification of patients with COPD with poor prognosis and implementation of proactive palliative care. *BMC Pulm Med*. 2014;14(1):54. doi:10.1186/1471-2466-14-54.

9. Leiva-Fernández J, Vázquez-Alarcón RL, Aguiar-Leiva V, Lobnig-Becerra M, Leiva-Fernández F, Barnestein-Fonseca P. Efficacy of an educational intervention in primary health care in inhalation techniques: study protocol for a pragmatic cluster randomised controlled trial. *Trials*. 2016;17(1):144. doi:10.1186/s13063-016-1269-5.

10. Fan VS, Gaziano JM, Lew R, et al. A comprehensive care management program to prevent chronic obstructive pulmonary disease hospitalizations: a randomized, controlled trial. *Ann Intern Med*. 2012;156(10):673-683. doi:10.7326/0003-4819-156-10-201205150-00003.

11. Randomized a, Trial C, Fan VS, et al. Original Research A Comprehensive Care Management Program to Prevent Chronic Obstructive Pulmonary Disease Hospitalizations. 2012;156(10).

12. Horton R, Rocker G, Dale A, Young J, Hernandez P, Sinuff T. Implementing a palliative care trial in advanced COPD: a feasibility assessment (the COPD IMPACT study). *J Palliat Med*. 2013;16(1):67-73. doi:10.1089/jpm.2012.0285.

13. Bove DG, Overgaard D, Lomborg K, Lindhardt BØ, Midtgaard J. Efficacy of a minimal home-based psychoeducative intervention versus usual care for managing anxiety and dyspnoea in patients with severe chronic obstructive pulmonary disease: a randomised controlled trial protocol. *BMJ Open*. 2015;5(7):e008031. doi:10.1136/bmjopen-2015-008031.

14. Ho T-W, Huang C-T, Chiu H-C, et al. Effectiveness of Telemonitoring in Patients with Chronic Obstructive Pulmonary Disease in Taiwan-A Randomized Controlled Trial. *Sci Rep*. 2016;6(March):23797. doi:10.1038/srep23797.

15. Booth S, Moffat C, Farquhar M, Higginson IJ, Burkin J. Developing a breathlessness intervention service for patients with palliative and supportive care needs, irrespective of diagnosis. *J Palliat Care*. 2011;27(1):28-36. http://www.ncbi.nlm.nih.gov/pubmed/21510129. Accessed December 21, 2017.

16. Farquhar MC, Prevost AT, McCrone P, et al. The clinical and cost effectiveness of a Breathlessness Intervention Service for patients with advanced non-malignant disease and their informal carers: mixed findings of a mixed method randomised controlled trial. *Trials*. 2016;17(1):185. doi:10.1186/s13063-016-1304-6.

17. Buckingham S, Kendall M, Ferguson S, et al. HELPing older people with very severe chronic obstructive pulmonary disease (HELP-COPD): mixed-method feasibility pilot randomised controlled trial of a novel intervention. *NPJ Prim care Respir Med*. 2015;25(November 2014):15020. doi:10.1038/npjpcrm.2015.20.

1. Thoonsen B, Vissers K, Verhagen S, et al. Training general practitioners in early identification and anticipatory palliative care planning: a randomized controlled trial. *BMC Fam Pract*. 2015;16(1):126. doi:10.1186/s12875-015-0342-6.

2. Teno J, Lynn J, Wenger N, et al. Advance directives for seriously ill hospitalized patients: Effectiveness with the patient self-determination act and the SUPPORT intervention. *J Am Geriatr Soc*. 1997;45(4):500-507. doi:10.1111/j.1532-5415.1997.tb05178.x.

3. Houben CHM, Spruit M a, Wouters EFM, Janssen DJ a. A randomised controlled trial on the efficacy of advance care planning on the quality of end-of-life care and communication in patients with COPD: the research protocol. *BMJ Open*. 2014;4(1):e004465. doi:10.1136/bmjopen-2013-004465.

4. Guell MR, de Lucas P, Galdiz JB, et al. [Home vs hospital-based pulmonary rehabilitation for patients with chronic obstructive pulmonary disease: a Spanish multicenter trial]. *Arch Bronconeumol*. 2008;44(10):512-518.

5. Cockcroft A, Bagnall P, Heslop A, et al. Controlled trial of respiratory health worker visiting patients with chronic respiratory disability. *Br Med J*. 1987;294(6566):225-228. http://www.scopus.com/inward/record.url?eid=2-s2.0-0023066589&partnerID=40&md5=3e93d1fa907c923524dc10325a85b276.

6. Higginson IJ, Bausewein C, Reilly CC, et al. An integrated palliative and respiratory care service for patients with advanced disease and refractory breathlessness: A randomised controlled trial. *Lancet Respir Med*. 2014;2(12):979-987. doi:10.1016/S2213-2600(14)70226-7.

7. Weber C, Stirnemann J, Herrmann FR, Pautex S, Janssens J-P. Can early introduction of specialized palliative care limit intensive care, emergency and hospital admissions in patients with severe and very severe COPD? a randomized study. *BMC Palliat Care*. 2014;13(47):1-7. doi:10.1186/1472-684X-13-47.

8. Duenk RG, Heijdra Y, Verhagen SC, Dekhuijzen RP, Vissers KC, Engels Y. PROLONG: a cluster controlled trial to examine identification of patients with COPD with poor prognosis and implementation of proactive palliative care. *BMC Pulm Med*. 2014;14(1):54. doi:10.1186/1471-2466-14-54.

9. Leiva-Fernández J, Vázquez-Alarcón RL, Aguiar-Leiva V, Lobnig-Becerra M, Leiva-Fernández F, Barnestein-Fonseca P. Efficacy of an educational intervention in primary health care in inhalation techniques: study protocol for a pragmatic cluster randomised controlled trial. *Trials*. 2016;17(1):144. doi:10.1186/s13063-016-1269-5.

10. Fan VS, Gaziano JM, Lew R, et al. A comprehensive care management program to prevent chronic obstructive pulmonary disease hospitalizations: a randomized, controlled trial. *Ann Intern Med*. 2012;156(10):673-683. doi:10.7326/0003-4819-156-10-201205150-00003.

11. Randomized a, Trial C, Fan VS, et al. Original Research A Comprehensive Care Management Program to Prevent Chronic Obstructive Pulmonary Disease Hospitalizations. 2012;156(10).

12. Horton R, Rocker G, Dale A, Young J, Hernandez P, Sinuff T. Implementing a palliative care trial in advanced COPD: a feasibility assessment (the COPD IMPACT study). *J Palliat Med*. 2013;16(1):67-73. doi:10.1089/jpm.2012.0285.

13. Bove DG, Overgaard D, Lomborg K, Lindhardt BØ, Midtgaard J. Efficacy of a minimal home-based psychoeducative intervention versus usual care for managing anxiety and dyspnoea in patients with severe chronic obstructive pulmonary disease: a randomised controlled trial protocol. *BMJ Open*. 2015;5(7):e008031. doi:10.1136/bmjopen-2015-008031.

14. Ho T-W, Huang C-T, Chiu H-C, et al. Effectiveness of Telemonitoring in Patients with Chronic Obstructive Pulmonary Disease in Taiwan-A Randomized Controlled Trial. *Sci Rep*. 2016;6(March):23797. doi:10.1038/srep23797.

15. Booth S, Moffat C, Farquhar M, Higginson IJ, Burkin J. Developing a breathlessness intervention service for patients with palliative and supportive care needs, irrespective of diagnosis. *J Palliat Care*. 2011;27(1):28-36. http://www.ncbi.nlm.nih.gov/pubmed/21510129. Accessed December 21, 2017.

16. Farquhar MC, Prevost AT, McCrone P, et al. The clinical and cost effectiveness of a Breathlessness Intervention Service for patients with advanced non-malignant disease and their informal carers: mixed findings of a mixed method randomised controlled trial. *Trials*. 2016;17(1):185. doi:10.1186/s13063-016-1304-6.

17. Buckingham S, Kendall M, Ferguson S, et al. HELPing older people with very severe chronic obstructive pulmonary disease (HELP-COPD): mixed-method feasibility pilot randomised controlled trial of a novel intervention. *NPJ Prim care Respir Med*. 2015;25(November 2014):15020. doi:10.1038/npjpcrm.2015.20.
