# Supplementary material for: Development of a complex intervention for early integration of palliative home care into standard care for end-stage COPD patients: A Phase 0–I study
Source: PLoS One. 2018 Sep 19;13(9):e0203326. doi: 10.1371/journal.pone.0203326 (PMC6145576; doi:10.1371/journal.pone.0203326)
Supplement: S3 Table — (DOCX) [file pone.0203326.s003.docx]

**S3 Table: Explorative literature search on interventions and intervention protocols**

| **Author,**  **Year, Country,**  **Journal** | **Main objectives** | **Design** | **Participants (n)** | **Inclusion criteria** | | **Components intervention** | **Primary/secondary**  **outcomes (PO/SO)** |
| --- | --- | --- | --- | --- | --- | --- | --- |
| **A. Completed trials** | | | | | | | |
| (Outcomes marked with *: significant outcomes; marked with ^#^: negative significant outcomes; marked with ^£^: non-significant outcomes but efficient intervention model; marked with ^&^: non-significant outcomes but inefficient intervention model) | | | | | | | |
| 1. Thoonsen et al  2015  The Netherlands  BMC Family Practice | Training for general practitioners (GPs) in identifying patients in need of palliative care and in structuring anticipatory palliative care planning in order to recognise patients in need of palliative care in a timely way and to structure anticipatory care. | Randomised controlled cluster (RCT) | GPs  Intervention: n= 80  Control: n= 79 | | All GP practices in two regions of the comprehensive cancer centre of the Netherlands were invited by mail to participate. GPs were excluded if they were a consultant in a palliative care team. | The intervention group: GPs were trained in identifying patients in need of palliative care and anticipatory care planning and for each identified patient, they were offered a coaching session with a specialist in palliative care to fine-tune a structured care plan  Control group:  Usual care | PO:  **^#^**the number of contacts with the out-of-hours GP cooperative  SO: *contacts with their own GP, *place of death and *hospitalisations in the last months of their life |
| 2. Ho et al  2016  Taiwan  Scientific reports | Investigate the effectiveness of telemonitoring in improving COPD patient outcomes. | RCT | COPD patients Intervention: n=53  Control: n=53 | | COPD patients > 20 years + COPD exacerbation as the main diagnosis, current or former smokers, spirometry-confirmed airflow limitation (FEV1), discharge to home, and accessibility to the internet and phone. | The telemonitoring group: patients had to report their symptoms daily using an electronic diary.  Control group:  Usual care | PO: **^#^**time to first re-admission for COPD exacerbation within six months of discharge.  SO: (1)* the time to first emergency room visit for COPD exacerbation, (2)* the number of all-cause hospital re-admissions, and (3)* the number of all-cause emergency room visits. |
| 3. Guëll et al  2015  Spain  Archivos de Bronconeumologia | To compare the effects of a simple home pulmonary rehabilitation program and an intensive hospital-based program in terms of the exercise tolerance and health-related quality of life (HRQOL) of patients with  COPD. | Prospective multi  center trial | COPD patients  Hospital group: n=28  Home group: n= 23 | | Patients with severe or very severe COPD: age between 50 and 75 years, classification as an ex-smoker or smoker intending to quit, forced expiratory volume in 1 second (FEV1) between 30% and 50% of reference, and stable condition free of exacerbations in the last 4 weeks. | 1 group received hospital and 1 group received home pulmonary rehabilitation.  Patients in both groups attended 2 informative sessions about the disease and 4 physical therapy sessions.  Patients in the hospital group then carried out a structured exercise program while home group patients performed low intensity exercises at home without supervision. | **^#^**Respiratory muscle function, arm strength, **^#^**leg strength (same results),  *Health-related quality of life (only on emotional function higher for hospital group) |
| 4. S. Fan et al  2012  United States  Annals of Internal Medicine | To determine the efficacy of a comprehensive care-management program (CCMP) in reducing the risk for COPD hospitalisation. | RCT | COPD patients: n=426 | | Patients had been hospitalised for COPD in the 12 months before enrollment. Additional inclusion criteria were a post-bronchodilator ratio of FEV1 to FVC below 0.70 with an FEV1 below 80% predicted, age older than 40 years, current or past history of cigarette smoking (10 packs/years), at least 1 visit in the past year to either a primary care or pulmonary clinic at a medical center, no COPD exacerbation in the past 4 weeks, ability to speak English, and access to a telephone. | Intervention group: COPD education during 4 individual and 1 group session, an action plan for identification and treatment of exacerbations, and scheduled proactive telephone calls for case management, based on the PRECEDE/PROCEED model of health program planning and evaluation.  Control group: Guideline based care | PO: **^#^**time from randomization to first COPD hospitalisation.  SO: *all-cause mortality, number of exacerbations, health-related quality of life, patient satisfaction, COPD-related knowledge, and self-efficacy. |
| 5. Cockcroft et al  1987  United Kingdom  British Medical Journal | To evaluate the role of respiratory health workers. | RCT | COPD patients: n=75 | | Chronic respiratory disability caused mainly by chronic obstructive airways disease. Admitted to hospital at least twice during the previous three years and new patients who had been seen within the past year. | Intervention group: was visited monthly by a respiratory nurse, who gave education and support, focusing on health rather than on disease, and varying in content according to individual needs. They followed a model identifying problems in activities of daily living and setting goals to increase independence in these activities.  Control group: care as usual | PO: *quality of life (by questionnaires).  SO: number and duration of admissions to hospital, and *the number of deaths. |
| 6. Higginson et al  2014  United Kingdom  The Lancet Respiratory Medicine | The effectiveness of early palliative care integrated with respiratory services for patients with advanced disease and refractory breathlessness. | Single blind RCT (1:1) | Patients with advanced disease Intervention: n=53  Control: n =52 | | Refractory breathlessness on exertion or rest (MRC dyspnoea scale score ≥2), despite optimum treatment of the underlying disease, as deemed by the identifying clinician; advanced disease such as cancer, COPD, CHF, ILD and motor neuron disease; willing to engage with short term home physiotherapy and occupational therapy; and able to provide informed consent. | Intervention group: a breathlessness support service: a short-term, single point of access service integrating palliative care, respiratory medicine, physiotherapy, and occupational therapy.  Control group: usual care | PO:* patient-reported breathlessness mastery at 6 weeks, a quality of life domain in the Chronic Respiratory Disease Questionnaire.  SO: severity of breathlessness on exertion in the previous 24 h, breathlessness, fatigue, and emotional function, quality of life, palliative needs, depression and anxiety and spirometry**.**  *Patient survival rate as a safety secondary endpoint. |
| 7.Buckingham et al  2015  United Kingdom  Npj Primary Care Respiratory Medicine | To identify and address the holistic care needs of people with severe COPD | 6 month pilot RCT  (ratio 3:1)  and interviews | Patients with severe COPD  Intervention: n=24  Control: n= 8 | | People registered with Lothian general practices admitted to Edinburgh Royal Infirmary or St John’s Hospital, Livingstone with a primary diagnosis  of exacerbation of COPD. | Intervention group: a proactive holistic assessment of physical, psychological, social and spiritual needs delivered by a specialist respiratory nurse shortly after an  admission with an exacerbation of COPD (HELP-COPD).  Control group: usual care | PO:* Functional assessment  SO: *health related quality of life, *physical, *psychosocial, spiritual well-being |
| 9. Teno et al  1997  United States  Journal of American Geriatric  Society | To assess the effectiveness of written Advance DirectiveS (ADS) in the care of seriously ill, hospitalised patients. In particular, to conduct an assessment after ADS were promoted by the Patient Self-Determination Act (PSDA) and enhanced by the effort to improve decision-making in the Study to Understand Prognoses and Preferences for Outcomes and Risks of Treatments (SUPPORT), focusing upon the impact of ADS on decision-making about resuscitation | Observational cohort study, RCT | Patients with severe illness.  Intervention: n= 2652  Control: n= 2152. | | Advanced stages of a diseases, such as COPD. | The intervention gave healthcare providers patient-specific prognoses for survival and function, and information about each patient’s and surrogate’s preferences, including whether the patient had written an advance directive. A nurse clinician specialist placed this information in the medical record or gave it to the attending physician. The nurse also facilitated communication among the patient, the surrogate, and the physician through education and empowering patients and family, arranging meetings, establishing  telephone contact, and other techniques to promote decision making that reflected the patients’ informed preferences. | *Patients' preferences for  pattern of care and for ventilator use; *symptoms; *life sustaining interventions; *and survival prognoses. |
| 10. Farquhar et al  2016  United Kingdom  BioMed Central | A search to establish the effectiveness and cost effectiveness of the Breathlessness Intervention Service (BIS) in advanced non-malignant conditions. | Single-centre phase III fast-track single-blind mixed method RCT | Breathless patients with non-malignant conditions Intervention: n= 44 Control: n= 43  And their carers. | | BIS referral criteria (diagnosed appropriately treated cause of breathlessness, were troubled by breathlessness in spite of optimization of underlying illness, and might benefit from a self-management programme) | Intervention group: BIS, multi-disciplinary complex intervention, underpinned by a palliative care approach, utilizing evidence-based non-pharmacological and pharmacological interventions to support patients.  Control group: standard care | PO: **^£^**0–10 numeric rating scale for patient distress due to breathlessness at four weeks. SO: **^&^**Chronic Respiratory Questionnaire, **^&^**Hospital Anxiety and Depression Scale, **^&^** Client Service Receipt Inventory, **^&^**EQ-5D and topic-guided interviews. |
| 11. Horton et al  2013  Canada  Journal of Palliative Medicine | To determine the feasibility of: 1) implementing a customized home-based palliative care service for patients and caregivers living with advanced COPD and 2) measuring outcomes of providing such services. | Single-centre, prospective, cohort observational study | COPD patients: n=33 Caregivers: n=18 | | Severe COPD: Severe shortness of breath resulting in the patient being too breathless to leave the house; or breathlessness after dressing/undressing (MRC dyspnea score of 5); or the presence of chronic respiratory failure (PaCO2 > 45); or clinical signs of right heart failure OR Moderate COPD: Shortness of breath causing the patient to stop walking after 100 meters or a few minutes on the level (MRC score of 3–4) and at least one the following: BMI < 21; FEV1 < 30% predicted; one or more hospital admissions for acute exacerbation of COPD in the previous year. | Intervention group: COPD self-management educational program based on the Living Well with COPD program plus the eighth educational module on EOL care and decision making delivered in the patient’s home over four to eight weeks. Comprehensive palliative care consultation within four weeks of completing educational component. Establish common medical chart in the home to facilitate coordinated care and sharing of information between various caregivers. Ongoing case management. | **^&^**Health-related quality of life (HRQoL), **^&^**caregiver burden symptom severity, *patient/ caregiver; satisfaction, utilization of acute care services, end-of-life (EOL) outcomes. |

| **Author,**  **Year, Country,**  **Journal,** | **Main**  **research question** | **Design** | **Participants (n)** | **Inclusion criteria** | **Components** | **Primary/secondary**  **Outcomes (PO/SO)** |
| --- | --- | --- | --- | --- | --- | --- |
| **B. Trial protocols** | | | | | | |
| 1. Fernandez et al.  2016  Spain  BioMed Central | To evaluate the efficacy of an educational intervention to train GPs in the right inhalation technique for the most commonly used inhalers. | Pragmatic cluster RCT | GPS  Intervention: n=10 Control: n=10  COPD patients:  n= 267  (10 patients/GP) | GPs: they must be doctors who care for patients included in the COPD PAI, use prescribed inhalation therapy and have consented to participate.  Patients: COPD diagnosis, being treated at the primary care centers included in the trial, being prescribed inhalation therapy and consenting to participate in the trial by signing the informed consent form. | The first level is patients with COPD who agree to participate in the trial and receive the educational intervention from their GPs. The second level is GPs who are primary health care professionals and receive the educational intervention. Intervention (for patients & GPs) is one session of the educational intervention with a monitor given to GPs for training in the right inhalation technique.  Control group: giving care as usual | PO: correct inhalation  technique in patients.  SO: functional status (spirometry) and quality of life. |
| 2. Weber et al  2014  Switzerland  BMC Palliative Care | To determine the effectiveness of the introduction of specialised palliative care on hospital, intensive care unit and emergency admissions of patients with severe and very severe COPD. | 3 year single centre RCT | COPD patients Intervention: n=90 Control: n= 90 | Patients with COPD  defined according to GOLD criteria with a stage III or IV disease and/or long-term treatment with domiciliary oxygen and/or home mechanical ventilation and/or one or more hospital admissions in the previous year for an acute exacerbation. | Intervention group: early palliative care consultation is added to standard care:  1. symptom management  2. understanding of illness and coping with the disease  3. anticipation  4. relatives support  5. social support  6. spiritual support  7. coordination of the health professionals  Control group: standard care only | PO: decrease in intensive care unit and emergency admissions.  SO: mood and anxiety of patients, quality of life, use of antibiotics during the last three months, completion of advance directives, documented  preferences for resuscitation, or nomination of surrogate decision maker will. |
| 3. Bausewein et al  2012  United Kingdom  BMC Pulmonary Medicine | To assess the effect of the Breathlessness Support Service (SSS), a multi- professional and inter- disciplinary outpatient service: on patients’ mastery  of breathlessness and other breathlessness measures; on physiological outcome measures; on caregiver burden; on patients’ experiences. To compare NHS resource usage and costs in the two study arms and to explore patients’ experiences using the BSS. | RCT (fast track),  face-to-face interviews with patients for outcome measure | Patients (unknown n) | Suffering from breathlessness on exertion or at rest due to advanced disease such as cancer, COPD, CHF, interstitial lung disease, ILD or MND^[[1]](#footnote-1)^.  Underlying disease should be optimally medically managed. Patients must be able to engage with short term physiotherapy. If patients are suffering from acute exacerbations they are put on a waiting list for two weeks and are then entered into the trial. | Intervention group: immediate access to BSS in addition to standard care.  Control group: standard best practice and access to BSS after six weeks. | PO: patients' improvement of mastery of breathlessness after six weeks assessed Respiratory Disease Questionnaire (CRQ)  SO: breathlessness severity, symptom burden, palliative care needs, service use, and respiratory measures (spirometry). |
| 4. Bove et al  2015  Denmark  BMJ Open | The efficacy of a minimal home-based psycho-educative intervention versus usual care for patients with COPD. | Single centre RCT | COPD patients (unknown n) | Patients with a confirmed COPD diagnosis, classified as category C or D (GOLD), HADS-A subscale score of ≥8 and were willing to participate and able to provide written consent. | Intervention group: psycho-educative session in the patient’s home in combination with a telephone booster session. Patients learn to interpret and react to physical and psychological symptoms that are related to dyspnea and associated anxiety.  Control group: usual care. | PO: patient reported anxiety as assessed by the HADS. |
| 5. Duenk et al  2014  The Netherlands  BMC Pulmonary Medicine | The primary objective is to assess the effects of proactive palliative care delivered by a specialised  palliative care team on the wellbeing of patients with COPD with poor prognosis and their informal caregivers. The secondary objective is to assess survival rate in COPD patients with proactive palliative care integrated with standard care versus standard care only. | Prospective cluster controlled trial (6 hospitals) with pre- and post-test. | COPD patients and main informal caregivers | Inclusion of hospitals: if there is a palliative care team.  Inclusion of patients: in hospitalisation for an acute exacerbation and ≥18 years old. | Intervention group: members of the specialised palliative care teams will receive a special training in the provision of proactive palliative care for patients with COPD. Patients in the intervention condition who are assigned for proactive palliative care will meet with a member of the specialised palliative care team within one week after enrollment and at least monthly thereafter in the outpatients setting for at least one year or until death. The main informal caregiver of the patient will be asked to be present at those meetings  Control group: standard care for COPD patients given by their treating lung specialists. | PO: change in quality of life (St George Respiratory Questionnaire (SGRQ) of the patient 3 months after inclusion.  SO: patient-related a) change in quality of life (SGRQ) of the patient 6, 9 and 12 months after inclusion; b) change in quality of life at the end of life (McGill QOL) 3, 6, 9 and 12 months after inclusion; c) change in psychological wellbeing (HADS) 3, 6, 9 and 12 months after inclusion; d) change in illness understanding 3, 6, 9 and 12 months after inclusion; e) number and length of unexpected hospital admissions; f) number and length of unexpected ICU admissions; g) are the choices of advance care planning documented in the medical file? (when yes/when no); h) place of death (ICU/hospital/hospice/nursing home/at home); i) is preferred place of death known? (when yes/when no); j) has this wish come true? (when yes/when no); k) length of survival of COPD patients with proactive palliative care integrated with standard care versus standard care only.  SO: informal caregiver-related:  a) change in informal caregiver burden (SPPIC) 3, 6, 9 and 12 months after inclusion; b) change in psychological wellbeing (HADS) at 3, 6, 9 and 12 months after inclusion; c) change in illness understanding at 3, 6, 9 and 12 months after inclusion. |
| 6. Houben et al  2014  The Netherlands  BMJ Open | To explore whether and to what extent structured advance care planning by a trained nurse, in collaboration with the chest physician, can improve outcomes in Dutch patients with COPD and their family. | A multicentre cluster RCT | Patients with severe to very severe COPD. | 1: a diagnosis of severe-to-very severe COPD (GOLD grade III or IV); 2: Discharged after hospital admission for a COPD exacerbation; 3: at least one family member, who will participate in the study. | Intervention group: structured advance care planning: reflection on patient’s goals, values and beliefs. Understanding the current and future medical situation, possible treatments and outcomes. Understanding life-sustaining treatments. Determining wishes regarding the current and future care. Encouraging discussions on advance care planning with healthcare providers and loved ones. Appointment of a surrogate decision-maker  Control group: standard care | PO: Quality of communication about end-of-life care; (HADS) For patients who died during the study period: quality of end-of-life care and quality of death and dying.  SO: concordance between patient’s preferences for end of-life care (patient’s preferences for CPR and mechanical ventilation; end-of-life preferences interview and received end-of-life care); psychological distress in bereaved family members of deceased patients with COPD; Inventory of Complicated Grief. |

1. Chronic obstructive pulmonary disease (COPD), chronic heart failure (CHF), interstitial lung disease (ILD) or motor neurone disease (MND). [↑](#footnote-ref-1)
