# Supplementary material for: Development of a complex intervention for early integration of palliative home care into standard care for end-stage COPD patients: A Phase 0–I study
Source: PLoS One. 2018 Sep 19;13(9):e0203326. doi: 10.1371/journal.pone.0203326 (PMC6145576; doi:10.1371/journal.pone.0203326)
Supplement: S4 Table — (DOCX) [file pone.0203326.s004.docx]

**S4 Table: Overview of barriers and facilitators on inclusion criteria and components from the expert consultations**

| **COMPONENTS** | **FACILITATORS*** | **BARRIERS*** |
| --- | --- | --- |
| **Advance Care Planning** | **ACP and palliative care (PC)**   - If you meet the criteria for ACP, you meet the criteria for (early) PC too (20, 22) - ACP is the perfect facilitator to start with (early) palliative care, beyond the terminal phase (18) - ACP should be a part of PC and curative care, and can integrate both (4) - You can start ACP independently from PC (13)   **Professional caregivers**   - Education/training about ACP for professional caregivers can help them start the conversations (3, 4, 12, 15, 19) - Training for professional caregivers in recognizing PC needs is useful for ACP (20) - GPs are well positioned to start ACP and then refer the patient to the PC team (3, 4, 5, 13, 17, 19, 22) - If the professional caregiver is feeling comfortable talking about ACP and PC, the patient is too (17) - Pulmonologists should be involved in ACP conversations too (4, 13) - An ACP-educator (e.g. a nurse) can start ACP conversations with every patient who gets diagnosed with COPD(12)   **Content and practical issues related to ACP**   - ACP should be an ongoing, gradual process, starting early in the disease trajectory (4, 13, 15, 17) - ACP should be started independently of the disease trajectory, as COPD is unpredictable (4) - It is better to structure ACP and embed it in the current care for better applicability (13, 20) - ACP is only useful when taking into account the specific situation of each patient (4, 15) - ACP is good to start in the non-terminal phase for improved quality of life (1, 2, 3, 12) - After an hospital admission is a good moment to start ACP (3, 17, 19, 22) - Start the conversation with the patient by explaining clearly that this does not mean they will die within a month (19)   **Patient**   - Especially people with a weaker social context and support benefit from planning ahead (22) - Guidance for patient and professional caregiver about future issues and care needs (4, 11, 15, 19, 22) - It is a good way to provide control for the patient about future situations (15, 17) - It reduces anxiety for the patient (5) | **Professional caregiver**   - It is not easy for professional caregivers to do ACP as they lack skills (12, 21) - It is time consuming for professional caregivers (5) - It is not well included in the education programs of medicine in Belgium (3) - It is an optional course for GPs in Belgium, but not obliged (3) - Pulmonologists do not have the communication skills to do ACP (15) - GPs lack communication skills to do ACP (20)   **Content and practical issues related to ACP**   - Sometimes it is more direct planning instead of advance planning, if the situation requires it (22) - Unclear when to introduce ACP because of the unclear disease trajectory of COPD(3, 13) - When the focus is on documentation instead of the conversation, it becomes problematic (13) - It became a key performance indicator for professional caregivers, so professional caregivers started to do ACP too soon and incautious, scaring patients (21)   **Patient**   - If a patient is in a stable phase, it is hard to start ACP conversations as they do not want to think/talk about the future at that moment (3, 11) - Family and patient do not understand the risk of dying (12) |
| **Involvement of loved ones** | **Loved ones in the (PC) care for COPD patients**   - Because they are involved in the decision-making processes and will help making better decisions for the patient if they receive the information too (22) - People are more concerned about the impact of decisions on the family, rather than the content of the decision (21, 22) - If loved ones are not convinced about a care option for the patient, they will not allow it (21) - Taking care for a COPD patient can be a burden for loved ones, as they experience e.g. isolation, pressure to look after them 24/7. Better involvement of loved one can help finding relief for those burdens(16) - In between a moderate COPD and a very terminal COPD, early PC could be really helpful for the patients and whole family, as it is a very tough, long period to cope with (16) - PC in the form of involvement of loved ones would make the loved ones realize that COPD is life-threatening, and would thus make the loved ones more supportive towards the patient (12, 9)   **Content**   - Mapping the family structure and involving the loved ones in order to provide the best PC, adjusted to the specific situation (15) - Loved ones can discuss together with the patient the best care options for both of them (15) | - Partners often do not realize the seriousness of the disease and force the patient to do things they are not able to do anymore (9) - Loved ones sometimes continue smoking despite the disease of their partner (9, 22) - The longer the disease takes, the more loved ones stop their involvement eventually (16) |
| **Knowledge and disease-insight of the patient** | **Professional caregiver**   - A lot depends on the way information is given (22) - GPs should inform the patient before they are introduced to the PC team (1 & 2)   **Content and practical issues**   - Repeatedly explain what is said over an extended period, so patients remember, realize and understand it (15) - Group meetings with other (severely-ill) COPD patients to talk about their disease and the problems that can arise (15, 22) - Info brochures with information about COPD (10)   **Knowledge, disease insight and PC**   - The content of PC can be explaining the patient that COPD is a terminal illness and which consequences this can have (11, 15, 17) - Explaining that COPD is a chronic, unstable illness, which is the trigger to start talking about the future (15) - Nurses can explain what PC is to the patients, with a focus on giving information (10)   **Patients**   - Patients need information (18) - Patients want information (15, 22) - If the patient understands its illness, they perform behavior which increases quality of life (f.ex. quit smoking) (9) | **Content and practical issues**   - Info-leaflets will not be read by patients as they are not used to it ( in Spain) (15) - Knowledge giving is more part of prevention strategy instead as a part of PC (15)   **Patient**   - Patients often have a low disease insight, which makes it harder to explain their situation (10) |
| **Pulmonary rehabilitation (PR)** | **Content**   - Replacing old therapies for respiratory therapy in the home care (9) - Less use of cortisone as it is bad for the muscles (3, 9) - Start rehabilitation on the moment of diagnosis (9) - Preventive work (9) - Focus on possible activities of daily living, degree of activity (9, 17, 18) - Focus on muscle strengthening of the legs or other parts of the body which improves metabolism of the oxygen, and makes the patient use oxygen more efficiently (1, 2, 3, 4, 18) - Focus on managing the condition (18) - Focus on body weight. The more weight you carry, the less easy to breathe (18) - Walking aids to improve stability encourages to be active and puts patients in a better position to breathe(18) - Blowing cool air on the face with a hand-held fan, improves the sensation of breathlessness and reduces panic as the nerve system is related to breathing center in the brain (18) - Teach patients breathing exercises, f.ex. to breathe out (3, 18, 21) - Giving very advanced patients little electroshocks to strengthen the legs if they cannot do anything else any more (18) - Relaxation exercises (1, 2) - Patient oriented, focused on the specific situation of each patient (21) - Teach patients to be more active (21)   **Pulmonary rehabilitation (PR) and (early) PC**   - If you do PR, you should be in a PC program at the same time, as there is clearly something very wrong with the patient (22) - You can do PR as a part of PC until the patient dies, if you adapt the impact of the therapy. Patients benefit from the smallest improvements in f.ex. breathlessness, level of activity (3, 18) - It would have a big and positive impact on the patient if PR would begin earlier in the PC (3, 9, 21) - PR should be worked out better in the PC home care, f.ex. home visits by physiotherapists and/or occupational therapists, otherwise the patient has to come each time to the hospital where PR is better (3, 18) - PR as a part of PC increases survival, gives a better quality of life, reduces symptoms and is effective as the patient can manage their own deterioration better(17, 18, 21) - PR can stabilize the patient in advance stages instead of further deterioration, which is an improvement (21) | **Content**   - Because PR doesn’t involve the loved ones, they will ultimately fail as a part of early PC, because you can’t ignore the family carers (21) - Sometimes PR is disease focused instead of patient focused, with general directives for all patients with the same disease, which has the risk of not treating the patient according to its own personal situation (21)   **Patients**   - Patients are used to their own physiotherapist and do not want to change that, even if they give the wrong therapy (9) - Patients are not willing to drive an extra mile for a better home physiotherapist (9) - If patients are too ill, they cannot or do not want to do any exercises or therapy anymore (18 21) - Patients do not understand the word rehabilitation (18) - If the family sees the patient being very breathless because of the exercises, they will stop this therapy for the patient as they do not want to see him/her suffering (21)   **Professional Caregivers**   - Only some physiotherapists have the knowledge of giving correct PR (9) - There might be insufficient focus on PR in graduate school for physiotherapy (9)   **Pulmonary rehabilitation and (early) PC**   - It is hard to predict when somebody should start with PR in early PC, because of the unclear disease trajectory (9) - There is insufficient funding to integrate PR in PC (17) |
| **Psychosocial support** | **Professional and informal caregivers**   - If volunteers will do the social support, they need some education on COPD and PC in case something would happen during the visits (16) - Volunteers can take over some tasks of the loved ones to continue the social support for the patient while relieving the loved ones from their care tasks (1, 2, 16) - Psychologists as a part of the palliative care team to help the patient with cognitive or behavioral approaches (14, 21) - Social workers as a part of the palliative care team to map the family structures, the main care, needs (14, 15) - Palliative nurses can take up the tasks of psychosocial support (1, 2) - It is better for the patient to stay at home, so the home network should be enlarged, while taking care of the family, assuring the patient they are looked after for (15)   **Content**   - Through classifications (e.g. ICF) is it possible to map the psychological and social needs of a PC patient (6) - Regular visits until the patients dies by volunteers (16) - Talking about basic topics like ‘how they are coping’ helps the patient to feel better (16) - Visits to the patient first one time in three months, but later at least every month, maybe every two weeks, depending on the situation of the patient (16) - For an intervention it might be better to have a strict criteria, let’s say one visit per month (16) - A relaxation CD to help the patient become calm (18) - Day programs with a fun and social theme, where the patient can participate in, for half a day or a full day (17) - Reduction of anxiety, as this is the main burden for the patient (5) - Talking/peer sessions for patients who suffer from f. ex oxygen dependency, with the biggest importance of the session is being together (10)   **Patients characteristics that indicate support is needed**   - One of the most important things for the patient, more than medical care support (14, 16) - Patients are afraid to leave their houses, and thus stay at home, alone (1, 2, 16) - Even during stable periods without exacerbations, they need the support (17) - Patients still lack sufficient psychosocial support , as the broader family do not know how to handle the sick family member (16) - Patients have the feeling their social circle does not understand themselves and they become isolated as a consequence (16) - The longer the diseases take, the less involved the family gets as it takes too long to cope with, while the patient needs them the most at those times (16) - The patient is often feeling lonely when they have no close relatives and just needs company (1, 2, 16, 17) - Advanced COPD patients are often between 50 and 70, most of them still have (many) friends, they just do not see them (16)   **Psychosocial support and (early) PC**   - Early PC is the perfect timing to start up the support (16) - The support, where caregivers are asking how the mood of the patient is, should be a part of the whole trajectory of the disease, starting from diagnosis, in curative care and continuing during (early) PC (17, 21) | **Psychosocial support and (early) PC**   - Current programs with support included in PC fail to reach the goal of aiding the patient (14) - It is good to start with the support as a part of early PC, but the biggest problem is that you don’t know how long the support will take (16) - If you start too early with the support as a part of early PC, it is not good. It is better to start when the patient is really advanced, when they can clearly understand why their functioning is very low (15) - If support would be needed during early PC, it would be because of the personality of the patient (15) - Psychosocial needs are not the big problem for these patients, it is very process-oriented and people seek solutions for it, e.g. not going by bike, but with the car(3) |
| **Self-management of patients** | **Content**   - Developing tools (e.g. a mantra, online tools) to manage their breathe (18) - Developing an online tool to help patients talk to their family and going through different situations (18) - Learning ways to let the patient cope with their exacerbations, f.ex. self-administration of medicines, relaxation exercises (1 & 2) - Self-management of breathlessness, as being out of breathe is the main reason why people run to the hospital (5, 22) - Info brochure for self-administering morphine (10) - Group meetings with other COPD patients to discuss self-help or self-management issues, combined with a fun and social factor (15, 17) - Group activities gives patients a better self-esteem as they feel they could do something fun for themselves (21) - Holistic self-management of the disease (5)   **Patients**   - If a patient can do more thing on their own, it decreases anxiety (21) | **Patients**   - Low-educated people are not used to the concept of self-control. They do not need self-control in the end stages of their lives/diseases, they just want to be healthy (15) - Contrary, high educated people can have (the feeling of) self-control in every stage of the disease, even if they have severe dyspnea (15) - Self-management is more important in the earlier stages of the disease (15) - Patients need help with everything, from washing to putting on clothes, to do their hair etc, so they can never reach (full) self-management (16) |
| **Symptom control** | **Content**   - Small doses of morphine for (subjective sense of) relief in breathlessness (1, 2, 3, 4, 10, 15, 17) - Short periods of medicines seem to work better for the patient than longer periods, as short periods of on-and-off medicines gives them the subjective feeling of control (17) - Control of breathlessness (18, 21) - Rescue teams doing acute symptom control in the home setting can reduce anxiety for the patients (11, 17) - Broad symptom control and full anamnesis focused on the patient itself (10, 18) - You need the same symptom control as used in hospital, but adapted to the home setting, f.ex. a mobile RT-BiPAP (17) - Assessment of emotional aspects of the patient and the family (15) - Using ESAS systematically during the first visit of the PC team (10)   **Symptom control and (early) PC**   - A patient with bad dyspnea or who cannot sleep at night cannot talk about the future too. So the first step is always symptom control (15, 18, 21) - Starting morphine earlier in the disease trajectory as a part of early PC makes it possible to adjust the doses better, as palliative nurses are more aware of the right amounts (3, 11) - After a rescue team does symptom control and decides whether a patient can stay at home or needs to go to the hospital, the PC team can start doing the further care for the patient (11, 17) - If symptom management happens before PC, there is a more clear view if the patient is in needs for PC or not (15) - Introducing PC is dependent on symptoms, different symptoms ask for different PC (10) - Calling the PC teams ‘symptom management experts’ or ‘symptom doctors’ and focusing the care on symptom management helps the patient to accept PC more (10, 12) |  |
| **INCLUSION CRITERIA** | **FACILITATORS*** | **BARRIERS*** |
| **After admission in hospital for exacerbation** | - An admission is a good starting point to ask the patient what they want to do if it happens again as they realize more the seriousness of their situation after this event (14, 20, 21, 22, 17) - After an admission there is quite high mortality in the following 12 months (22) - After second (or more) admission for exacerbation, as they realize more the seriousness of their situation after this (12, 17, 19, 21, 22) - After at least two admissions in the hospital during the last months, for eight months survival(14, 16) - It depends, if you do not want your patient to die during the intervention, you might need to go for inclusion after the first admission (22) | - After two or three admissions in the last month, the mortality rate of a patient is around six months (22) - Some patients can only start to talk about PC or further care after the 12^th^ exacerbation, so it depends on the personality of the patient (4) |
| **Functioning of the patient** | - Declining functionality in the last months (14) - Include the patient, based on its personal level of functioning (16) - If the patient becomes frail (22) - Using the CAT-questionnaire (10) - Using depression scales for inclusion (10) - MMSE score (mini mental state examination) (10) |  |
| **Depending on lung function test and prognosis tests** | - BODE-index 7 for eight months survival(14) - GOLD IV for eight months survival (14) - Results of a full quality of life assessment will indicate whether a patient is eligible for PC or not (16) - Walking speed is a good predictor of illness (18) - The six minute walking test with 30, 60, maybe up to 100 meter in 6 minutes is really slow and shows that the patient is in a bad state (18) - If lung function tests prove the patient is in a severe stage of the disease (22) - Prognosis tests with general criteria, not seen from the individual, as the intervention is limited in time for inclusion of patients (22) - The NNCSI, a care standard developed in the Netherlands which replaces the GOLD criteria (19) - There is nothing good enough, but BODE index can help the caregiver to see if there is something they can do for the patient (15) - The SPICT or the NECPAL let the caregivers think about if the patient will die within the following year (4) - Combination of GOLD criteria and diffusion test (12) | - Same results on FEV1 have different impacts on patients (9) - Depending on the social context of the patient, there are different implications for the patient (19) - GOLD III or IV indicate a worsening of symptoms and more exacerbations, but that does not mean patients become in need of PC (4) - A patient with GOLD III can be kept alive by PC teams, but not by a pulmonologist (19) - BODE index 5 patients do not have (sufficient) symptoms to start PC (15) - BODE index more than 5 is too late, depending on the patient they can have 6 to 12 months survival (15) - SPICT-tools and surprise questions are not useful as COPD is too unpredictable (16, 20) |
| **Social context** |  | - Difficult predictor or PC needs (16) |
| **Opinion of the caregiver** | - Why the caregiver thinks the patient should not be referred to hospital anymore (7) |  |
| **Being housebound** | - The moment the patient is housebound, they can have their first PC consult, as they are suffering in any case (12, 14, 16) | - When housebound, it is too late to start early PC (22) |
| **Oxygen dependency** | - If a patient becomes oxygen dependent (12, 22) |  |

*All numbers indicate which expert (who has received a number, see table 1 in manuscript) mentioned this barrier or facilitator.
